# Extended and standard duration weight-loss programme referrals for adults in primary care (WRAP): a randomised controlled trial

**DOI:** 10.1016/S0140-6736(17)30647-5

**Published:** 2017-06-03

**Authors:** Amy L Ahern, Graham M Wheeler, Paul Aveyard, Emma J Boyland, Jason C G Halford, Adrian P Mander, Jennifer Woolston, Ann M Thomson, Melina Tsiountsioura, Darren Cole, Bethan R Mead, Lisa Irvine, David Turner, Marc Suhrcke, Laura Pimpin, Lise Retat, Abbygail Jaccard, Laura Webber, Simon R Cohn, Susan A Jebb

**Affiliations:** a MRC Human Nutrition Research, Cambridge, UK; b MRC Epidemiology Unit, University of Cambridge, Cambridge, UK; c MRC Biostatistics Hub for Trials Methodology Research, University of Cambridge, Cambridge, UK; d Cancer Research UK and UCL Cancer Trials Centre, University College London, London, UK; e Nuffield Department of Primary Care Health Sciences, University of Oxford, Oxford, UK; f Department of Psychological Sciences, University of Liverpool, Liverpool, UK; g Norwich Medical School, University of East Anglia, Norwich, UK; h Centre for Health Economics, University of York, York, UK; i UK Health Forum, London, UK; j Department of Health Services Research and Policy, London School of Hygiene & Tropical Medicine, London, UK

## Abstract

**Background:**

Evidence exist that primary care referral to an open-group behavioural programme is an effective strategy for management of obesity, but little evidence on optimal intervention duration is available. We aimed to establish whether 52-week referral to an open-group weight-management programme would achieve greater weight loss and improvements in a range of health outcomes and be more cost-effective than the current practice of 12-week referrals.

**Methods:**

In this non-blinded, parallel-group, randomised controlled trial, we recruited participants who were aged 18 years or older and had body-mass index (BMI) of 28 kg/m^2^ or higher from 23 primary care practices in England. Participants were randomly assigned (2:5:5) to brief advice and self-help materials, a weight-management programme (Weight Watchers) for 12 weeks, or the same weight-management programme for 52 weeks. We followed-up participants over 2 years. The primary outcome was weight at 1 year of follow-up, analysed with mixed-effects models according to intention-to-treat principles and adjusted for centre and baseline weight. In a hierarchical closed-testing procedure, we compared combined behavioural programme arms with brief intervention, then compared the 12-week programme and 52-week programme. We did a within-trial cost-effectiveness analysis using person-level data and modelled outcomes over a 25-year time horizon using microsimulation. This study is registered with Current Controlled Trials, number ISRCTN82857232.

**Findings:**

Between Oct 18, 2012, and Feb 10, 2014, we enrolled 1269 participants. 1267 eligible participants were randomly assigned to the brief intervention (n=211), the 12-week programme (n=528), and the 52-week programme (n=528). Two participants in the 12-week programme had been found to be ineligible shortly after randomisation and were excluded from the analysis. 823 (65%) of 1267 participants completed an assessment at 1 year and 856 (68%) participants at 2 years. All eligible participants were included in the analyses. At 1 year, mean weight changes in the groups were −3·26 kg (brief intervention), −4·75 kg (12-week programme), and −6·76 kg (52-week programme). Participants in the behavioural programme lost more weight than those in the brief intervention (adjusted difference −2·71 kg, 95% CI −3·86 to −1·55; p<0·0001). The 52-week programme was more effective than the 12-week programme (−2·14 kg, −3·05 to −1·22; p<0·0001). Differences between groups were still significant at 2 years. No adverse events related to the intervention were reported. Over 2 years, the incremental cost-effectiveness ratio (ICER; compared with brief intervention) was £159 per kg lost for the 52-week programme and £91 per kg for the 12-week programme. Modelled over 25 years after baseline, the ICER for the 12-week programme was dominant compared with the brief intervention. The ICER for the 52-week programme was cost-effective compared with the brief intervention (£2394 per quality-adjusted life-year [QALY]) and the 12-week programme (£3804 per QALY).

**Interpretation:**

For adults with overweight or obesity, referral to this open-group behavioural weight-loss programme for at least 12 weeks is more effective than brief advice and self-help materials. A 52-week programme produces greater weight loss and other clinical benefits than a 12-week programme and, although it costs more, modelling suggests that the 52-week programme is cost-effective in the longer term.

**Funding:**

National Prevention Research Initiative, Weight Watchers International (as part of an UK Medical Research Council Industrial Collaboration Award).

## Introduction

The burden of disease attributable to excess bodyweight places considerable strain on health-care resources across the world.^1^, ^2^ Behavioural weight-management programmes are the first-line method to aid weight loss in people who are overweight or obese, and although there is good evidence that some programmes can be effective, other programmes have not been shown to be effective.^3^ This variation in effectiveness might be due to differences in content and format of interventions, including how long support is provided. In the UK, the National Institute for Health and Care Excellence public health guidance^4^ recommends that behavioural weight-management programmes should last a minimum of 12 weeks, which is the standard length of the most commonly commissioned interventions.^5^ However, there is conflicting evidence about whether longer treatment (>12 weeks) duration would be more effective.^6^, ^7^

**Research in context**

**Evidence before this study**

A systematic review and meta-analysis was done in November, 2012, to synthesise data from 37 trials of behavioural weight-management programmes delivered in a context that could be replicated in routine clinical practice. Three studies investigated primary care referral to a commercial open-group programme compared with a control group and pooled results showed a mean difference of 2·22 kg in favour of the intervention group, but no consistent effects on markers of cardiovascular risk. The quality of the evidence was assessed as moderate. These interventions lasted 12, 52, and 104 weeks with no studies comparing effectiveness with different durations of treatment. Indirect comparisons across all 37 studies found no effect of duration of intervention on weight loss at 12 months. However, findings from a previous systematic review and meta-analysis of direct comparisons between interventions of different lengths, mostly from controlled research studies, showed that interventions providing extended care led to 3·2 kg less weight regain than control interventions over a mean follow-up period of 17·6 months after initial weight loss. We did an updated search of PubMed and Scopus with search terms for “overweight” and “obesity”, “diet” and “exercise”, and “weight-loss interventions”, for papers published up until Jan 12, 2017, and found no new direct comparisons of treatment duration.

**Added value of this study**

We found that referral to a commercial, open-group behavioural programme for 12 weeks or 52 weeks resulted in more weight loss than a brief self-help intervention. This finding extends previous findings by showing that referral for 52 weeks achieves greater weight loss than standard 12-week referrals currently used in the UK National Health Service over 2 years. Contrary to common criticisms that these interventions could exacerbate health inequalities, there was no evidence that the outcome of treatment is affected by socioeconomic factors such as sex, education, and income. We also show, for the first time to our knowledge, that this extended referral achieves improvements in fasting glucose concentration and glycated haemoglobin equivalent to more intensive health professional-led interventions. Using microsimulation modelling, we show for the first time that, over a 25-year period, the 12-week programme is cost-saving compared with a brief intervention, and that the 52-week programme is cost-effective compared with the 12-week programme.

**Implications of all the available evidence**

Referral to a commercial open-group behavioural weight-loss programme for 12 weeks is an effective weight-loss intervention and could be cost-saving for adults in the general population in the long term. Extending the referral length from 12 weeks (UK standard) to 52 weeks could increase the clinical effectiveness of these programmes, by achieving increased weight loss and reductions in risk factors for diabetes and cardiovascular disease compared with the 12-week programme. Although the 52-week programme is more expensive in the short term, the programme is likely to be cost-effective in the longer term because of greater reductions in disease incidence.

Open-group, behavioural weight-loss programmes are among the most commonly commissioned programmes in the UK and evidence suggests that these programmes are both clinically effective and cost-effective.^3^, ^8^ We aimed to establish whether 52-week referral to an open-group weight-management programme would result in significantly greater weight loss and improvements in a range of secondary health outcomes in participants and be more cost-effective than the current practice of 12-week referrals.

## Methods

### Study design

This study was a multi-centre, non-blinded, multi-arm randomised controlled trial with imbalanced randomisation. The full protocol has been described elsewhere.^9^ Briefly, participants were recruited from 23 primary care practices in England between Oct 18, 2012, and Feb 10, 2014. Recruitment and follow-up was done in three research centres: Medical Research Council (MRC) Human Nutrition Research (Cambridge; coordinating centre), the University of Liverpool, and the University of Oxford. Research teams at Cambridge and Liverpool recruited local practices and research staff did study visits at the research centre. The research team at Oxford recruited practices across southern and eastern England and practice staff (usually a research nurse) did study visits at the practice.

Ethical approval was received from NRES Committee East of England Cambridge East and local approvals from NRES Committee North West Liverpool Central and NRES Committee South Central Oxford. This trial was registered with Current Controlled Trials, number ISRCTN82857232.

### Participants

Eligible participants (those aged 18 years or older and had a body-mass index [BMI] of 28 kg/m^2^ or higher) were identified through practice records. Exclusion criteria were planned (within 2 years) or current pregnancy; previous or planned bariatric surgery; current participation in a structured, monitored weight-loss programme; participation in other research that could confound outcome measures; eating disorders; and non-English speaking or special communication needs. Practices could exclude additional patients who they felt were inappropriate to invite, but were asked to report reasons for exclusion. Additional reasons for exclusion included terminal illness or palliative care, dementia, a severe mental health problem or learning difficulty, carer for a terminally ill relative, or recently bereaved. Patients were invited by letter and asked to contact the local study coordinator for telephone screening if they were interested in participating. Eligible and willing participants were given an appointment, during which a member of the research team weighed them and measured their height to confirm eligibility before randomisation. If more than one household member was eligible and interested in participating, the first to enrol was taken as the participant. All participants gave written informed consent.

### Randomisation and masking

Participant details were entered into the trial database, which randomly assigned participants with a valid BMI to have one of three interventions (brief intervention [given a booklet of self-help weight-management strategies], referral to the commercial, open-group, behavioural weight-loss programme [behavioural programme] for 12 weeks, or referral to the same programme for 52 weeks) in a 2:5:5 ratio stratified by centre and gender, with a block size of 12. The randomisation sequence was generated by the trial statistician at the time of protocol development using Stata (version 12.1) and programmed into the database by the data manager (DC). The sequence was unknown to research staff and participants. The database revealed the group allocation after participants were enrolled and entered into the database. Participants and research staff were not blinded to the intervention allocation after randomisation because of the nature of the intervention and the trial design.

### Procedures

Participants assigned to the behavioural programme were asked to attend a local Weight Watchers meeting once a week for the duration of their intervention (12 weeks or 52 weeks). At the baseline visit, participants were given a list of local meeting times and locations, a voucher booklet for 12 visits (the expiry date was set for 14 weeks from baseline), and a unique code to access digital tools for the duration of their intervention. Meeting vouchers were identical to those used in the National Health Service (NHS) referral schemes operating throughout the country and allowed participants to attend meetings without charge. At the meeting, participants were asked to give the voucher to the group leader, but were asked not to mention their participation in the trial to the group leader or other members. Participants assigned to the 52-week programme were given three additional books of vouchers when they returned for their 3-month visit (expiry date set for 54 weeks from baseline).

Participants allocated to the brief intervention were given a 32-page printed booklet by the British Heart Foundation of self-help weight-management strategies^10^ and research staff read a scripted introduction that drew attention to each section of the booklet.

All participants attended measurement appointments at baseline and 3, 12, and 24 months. Height was measured to the nearest 0·1 cm using a stadiometer. Weight and fat mass were measured to the nearest 0·1 kg using a 4-point segmental body composition analyser (Tanita, Amsterdam, The Netherlands). Waist circumference was measured to the nearest 0·1 cm with a tape measure, halfway between the lowest rib and the iliac crest. Blood pressure was measured three times in a seated resting state with an automated blood pressure monitor, and the mean calculated. Biochemical measurements were optional. Willing participants were asked to give a fasting blood sample at the baseline visit and 12 months for analysis of glucose concentration, glycated haemoglobin (HbA_1c_), and lipid profile. All samples were analysed in Cambridge using standardised methods (appendix p 2).

At each visit, participants self-reported their use of weight-loss methods, including the allocated intervention, and completed the EuroQol 5-dimension 3-level (EQ5D-3L) questionnaire^11^, ^12^ as a measure of quality of life. Data on health-care resource use was also self-reported. Participants who were unable or unwilling to attend a 12-month visit (primary outcome measurement) were asked to provide a self-measured weight by phone or email. Self-reported weights are not included in the primary outcome analysis, but are included in a sensitivity analysis (appendix p 3).

### Outcomes

The primary outcome was change in bodyweight at 12 months. The secondary clinical outcomes were bodyweight at 3 months and 24 months; proportion of participants losing 5% or more of baseline bodyweight or 10% or more of baseline bodyweight at 3, 12, and 24 months; waist circumference, fat mass, and blood pressure at 3, 12 and 24 months; fasting blood glucose concentration, HbA_1c_, triglycerides and HDL, LDL, and total cholesterol at 12 months; and self-reported quality of life (EQ5D-3L) and health resource use at 3, 12, and 24 months. We did not anticipate that adverse events related to the interventions would occur and so did not formally record these.

### Statistical analysis

We calculated the sample size based on data from our previous trials^13^, ^14^ with an expected difference of 2·3 kg between the brief intervention and combined behavioural programme groups, 1·3 kg difference between 12-week and 52-week programmes, and an assumed SD of 6 kg. The hierarchical closed-testing procedure was used to compare the behavioural programme groups with the brief intervention groups using a one-sided test and then, only if significant at the 5% level, we did a two-sided test for a difference between 12-week and 52-week programmes to preserve a type I error rate of 5% without the need for a multiplicity correction. With a sample of 1200 participants (200 participants in the brief intervention group, 500 participants in the 12-week programme, and 500 participants in the 52-week programme), we had 99·95% power to detect a difference of 2·3 kg between the brief intervention group and the behavioural programme groups, and 92·87% power to detect a difference of 1·3 kg between 12-week and 52-week programmes. The overall power of the study was 92·82%.

Analyses were prespecified in the published protocol.^9^ The primary analyses assessed differences between the intervention groups in mean weight change from baseline to 12 months. Because of levels of attrition commonly encountered in weight-loss trials, four analysis approaches were taken to account for the effect of missing data: a missing at random (MAR) analysis using a variance components model; a completers only analysis; baseline observation carried forward (BOCF); and last observation carried forward (LOCF). For the MAR analysis, we calculated mean weight loss and SE via a multiple imputation model using multivariate normal regression; we imputed 20 datasets for weight separately for each treatment group, with baseline weight, weight at 3 months, weight at 12 months, and weight at 24 months regressed on centre. A model for multivariate normal data with baseline weight, weight at 3 months, weight at 12 months, and weight at 24 months as the outcome was fitted using measured weights at each timepoint via generalised least squares, with intervention group, visit, intervention group-by-visit interaction, and centre included as fixed effects. For the participants who completed the study only, BOCF and LOCF analyses, fixed-effect models for continuous normal data were fitted to the 12-month weight data. The fixed effects were intervention group, centre, and baseline weight. We analysed secondary outcomes with the same regression-based models. Results reported in this Article used the MAR assumption. Results obtained using other assumptions are in the appendix (p 4). We did sensitivity analyses to examine whether findings were sensitive to timing of the 12-month assessment or inclusion of self-reported weights. All analyses were done in Stata (version 13.1).

A review^4^ highlighted the absence of evidence of this type of programme on socioeconomic inequalities, so we also did a post-hoc analysis of potential interactions between intervention effects and gender, educational qualification, and income. We calculated coefficient estimates for each fixed effect.

To establish within-trial cost-effectiveness over 24 months, we calculated the incremental cost-effectiveness ratio (ICER) as incremental cost per additional kg of weight loss (see appendix for details p 5). If the participant attended at least one session, the NHS would be charged a flat rate of £48·50 (12-week programme) or £190 (52-week programme). If they did not attend, there was no charge. We estimated non-intervention NHS costs from health-resource use questionnaires, which were completed by participants at baseline, 3, 12, and 24 months and were framed within a 3-month recall period.^15^ We used area under the curve methods to ascertain the full NHS costs incurred over the 24 months' follow-up period.^16^ We did a secondary analysis to examine the ICER over a 1-year time horizon to enable comparison with similar studies with shorter follow-up.

We used the microsimulation model developed for the Foresight: Tackling Obesities project^17^, ^18^ to estimate the effect of the three interventions on disease incidence, health-care costs, quality-adjusted life-year (QALY), and ICERs for 25 years following baseline (appendix p 14). We used mean change in BMI for each intervention at 1 year and 2 years for this analysis, and assumed that between 2 years and 5 years all participants returned to their baseline weight in a linear manner (ie, regained all weight lost) and followed national trends based on data derived from repeated cross-sectional samples in the Health Survey for England.^19^

The number of meetings attended by the participants in the behavioural programme groups between baseline and 3 months, 9 months, and 12 months, and 21 months and 24 months, was self-reported. The proportion of participants who self-reported use of other weight-loss interventions was also calculated.

Data management was overseen by the coordinating centre's data manager. The dataset linking treatment group to participant outcomes was only released to the investigators after completion of data collection for the primary outcome.

### Role of the funding source

The funders of the study had no role in the design, data collection, data analysis, data interpretation, or writing of the report. ALA and GMW had full access to all data and ALA had final responsibility for the decision to submit for publication.

## Results

**Figure 1:**
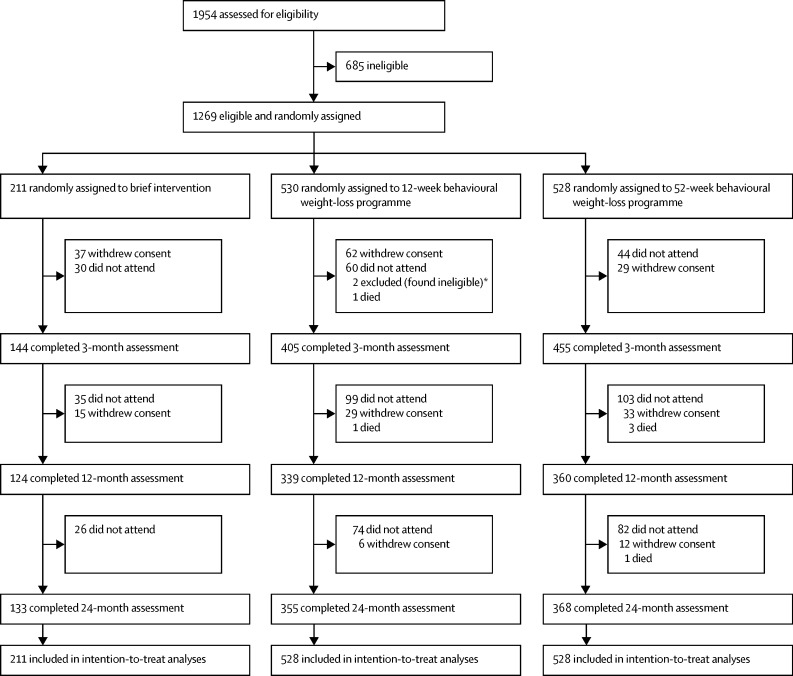
Trial profile *Excluded from intention-to-treat analyses.

Between Oct 18, 2012, and Feb 10, 2014, 1954 participants were screened and 1269 were eligible and were randomly allocated an intervention (figure 1). After the baseline appointment and before the interventions were started, two participants were excluded because their general practitioner reported illnesses that would have excluded them. These participants were removed and the remaining 1267 participants were included in the primary analyses. Data on participants who died during the trial are included up until the event. The number of participants completing each assessment was 1004 (79%) of 1267 participants at 3 months, 823 (65%) of 1267 participants at 12 months, and 856 (68%) of 1267 participants at 24 months. Using Pearson's χ^2^ test, there was a borderline significant association between the percentage of participants attending the 12-month appointment and intervention group (p=0·0475); no such association for participants completing the 24-month assessment was found (p=0·21). Examination of the effect of individual characteristics on attendance by intervention group showed no evidence of bias in attendance during the study (table 1). Analyses of biochemical risk factors are based on 837 (66%) of 1267 participants who provided a blood sample at baseline.

**Table 1:** Baseline characteristics of participants

		**Brief intervention (N=211)**		**12-week programme (N=528)**		**52-week programme (N=528)**	
		n or n (%)	Mean (SD)	n or n (%)	Mean (SD)	n or n (%)	Mean (SD)
Age (years)		211	51·9 (14·1)	528	53·6 (13·3)	528	53·3 (14·0)
Weight (kg)		211	96·1 (16·4)	528	96·6 (17·9)	528	95·7 (16·4)
Height (cm)		211	167 (9·5)	528	167 (8·9)	528	167 (9·0)
Body-mass index (kg/m^2^)		211	34·4 (4·6)	528	34·7 (5·4)	528	34·5 (5·1)
Fat mass (kg)		204	39·2 (9·9)	515	39·6 (11·8)	517	39·4 (11·1)
Waist circumference (cm)		210	110 (11·9)	528	111 (12·4)	528	110 (12·7)
Systolic blood pressure (mm Hg)		210	130·6 (15·7)	526	133·5 (17·2)	527	133·3 (18·1)
Diastolic blood pressure (mm Hg)		210	79·7 (9·2)	526	80·7 (9·7)	527	79·9 (10·0)
Fasting glucose (mmol/L)		134	5·8 (1·9)	345	5·6 (1·6)	326	5·8 (1·8)
HbA_1c_(mmol/mol)		143	41·9 (11·2)	354	40·9 (9·8)	338	41·7 (10·4)
HbA_1c_(%)		143	6·0 (1·0)	354	5·9 (0·9)	338	6·0 (0·9)
Triglycerides (mmol/L)		146	1·6 (0·9)	357	1·6 (0·8)	339	1·5 (0·7)
Cholesterol (mmol/L)		146	5·5 (1·2)	357	5·3 (1·1)	337	5·3 (1·1)
LDL cholesterol (mmol/L)		145	3·1 (1·2)	353	3·0 (1·0)	339	2·9 (1·0)
HDL cholesterol (mmol/L)		146	1·6 (0·6)	357	1·6 (0·6)	339	1·7 (0·6)
Quality of life (EQ5D-3L tariff)		197	0·786 (0·266)	508	0·783 (0·249)	504	0·793 (0·249)
Quality of life (EQ-Vas)		201	70·3 (18·9)	515	70·9 (18·0)	506	70·0 (19·9)
Sex							
	Female	143 (68%)	..	357 (68%)	..	359 (68%)	..
	Male	68 (32%)	..	171 (32%)	..	169 (32%)	..
Gross household income (per annum)							
	<£20 000	65 (31%)	..	125 (24%)	..	138 (26%)	..
	£20 000–39 999	56 (27%)	..	132 (25%)	..	137 (26%)	..
	≥£40 000	51 (24%)	..	132 (25%)	..	123 (23%)	..
	Missing or prefer not to say	39 (18%)	..	139 (26%)	..	130 (25%)	..
Ethnicity							
	Asian or Asian British	9 (4%)	..	11 (2%)	..	15 (3%)	..
	Black or black British	5 (2%)	..	12 (2%)	..	6 (1%)	..
	Mixed or multiple ethnic group	4 (2%)	..	4 (1%)	..	7 (1%)	..
	White or white British	181 (86%)	..	480 (91%)	..	475 (90%)	..
	Other	2 (1%)	..	6 (1%)	..	7 (1%)	..
	Missing or prefer not to say	10 (5%)	..	15 (3%)	..	18 (3%)	..
Education							
	Higher degree or equivalent	23 (11%)	..	79 (15%)	..	68 (13%)	..
	University degree or equivalent	48 (23%)	..	108 (20%)	..	97 (18%)	..
	Post-secondary education	10 (5%)	..	14 (3%)	..	10 (2%)	..
	A-levels or equivalent	53 (25%)	..	95 (18%)	..	110 (21%)	..
	GCSEs or equivalent	55 (26%)	..	153 (29%)	..	155 (29%)	..
	None	7 (3%)	..	25 (5%)	..	27 (5%)	..
	Missing or prefer not to say	15 (7%)	..	54 (10%)	..	60 (11%)	..

HBA_1c_=glycated haemoglobin A_1c_. EQ5D-3L=EuroQol 5-dimension 3-level. EQ-Vas=EuroQol Visual Analogue Scale. A-levels=advanced levels. GCSE=General Certificate of Secondary Education.

Participants had a mean BMI of 34·5 kg/m^2^ (SD 5·2) and had a mean age of 53·2 years (13·8). 859 (68%) of 1267 participants were female, 1136 (90%) were white, 171 (13%) had diabetes, 631 (50%) had hypertension.

**Figure 2:**
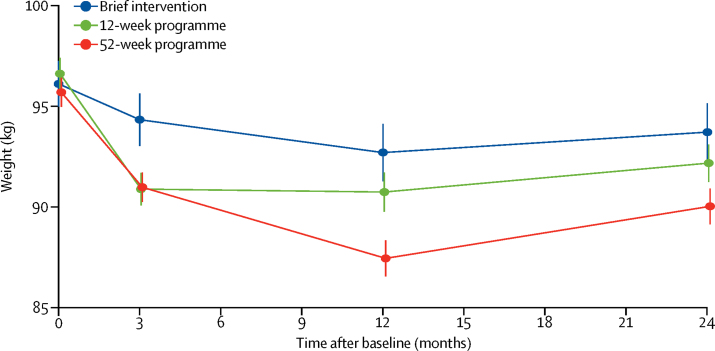
Bodyweight over 24 months of follow-up Data are mean of all measured weights at each timepoint (SE).

The weight trajectories of the three intervention groups at each timepoint using all measured weights are in figure 2. Mean weight change at 3 months was −2·04 kg (SE 0·30) after brief intervention, −4·84 kg (0·21) in the 12-week programme, and −4·62 kg (0·18) in the 52-week programme. Participants in the behavioural programme groups (12 weeks and 52 weeks combined) lost more weight than those in the brief intervention (adjusted difference −2·67 kg, 95% CI −3·28 to −2·07; p<0·0001] and there was no significant difference between the 12-week and 52-week programmes (0·22 kg, −0·26 to 0·69; p=0·371). The primary outcome of mean weight change at 12 months was −3·26 kg (SE 0·68) in brief intervention, −4·75 kg (0·35) in the 12-week programme, and −6·76 kg (0·42) in the 52-week programme (table 2). Participants in the combined behavioural programme groups lost significantly more weight at 12 months than those who had a brief intervention (−2·71 kg, −3·86 to −1·55; p<0·0001) and participants in the 52-week programme lost significantly more weight than those in the 12-week programme (−2·14 kg, −3·05 to −1·22; p<0·0001). Participants in all groups regained weight between 12 and 24 months, but the differences between groups remained significant. Weight change between baseline and 24 months was −2·30 kg (SE 0·73) in brief intervention, −3·00 kg (0·37) in the 12-week programme, and −4·29 kg (0·44) in the 52-week programme. Participants randomly assigned the behavioural programme in the 12-week and 52-week programme lost significantly more weight than those receiving a brief intervention (−1·44 kg, 95% CI −2·87 to 0·00; p=0·0247). Participants randomly assigned the 52-week programme lost significantly more weight than those who went on the 12-week programme (−1·32 kg, −2·46 to −0·18; p=0·0231; table 2).

**Table 2:** Weight change from baseline at 3, 12, and 24 months

	**Weight change from baseline, kg (SE)**			**Behavioural programme *vs* brief intervention (one-sided)**		**52-week programme *vs* 12-week programme (two-sided)**	
	Brief intervention	12-week programme	52-week programme	Adjusted difference (95% CI)	p value	Adjusted difference (95% CI)	p value
3 months	−2·04 (0·30)	−4·84 (0·19)	−4·62 (0·17)	−2·67 (−3·28 to −2·07)	<0·0001	0·22 (−0·26 to 0·69)	0·371
12 months	−3·26 (0·68)	−4·75 (0·35)	−6·76 (0·42)	−2·71 (−3·86 to −1·55)	<0·0001	−2·14 (−3·05 to −1·22)	<0·0001
24 months	−2·30 (0·73)	−3·00 (0·37)	−4·29 (0·44)	−1·44 (−2·87 to 0·00*)	0·0247	−1·32 (−2·46 to −0·18)	0·0231

Missing at random analysis with 20 imputed datasets (N=1267 at each timepoint). Treatment effects obtained from mixed-effects models with residuals structured as a first-order auto-regressive process stratified by treatment group. Adjusted differences are shown between combined treatment groups (12-week programme and 52-week programme) versus brief intervention (test 1) and 52-week programme versus 12-week commercial programme (test 2). Analyses are adjusted for baseline observation and centre.

* Rounded from <0·00.

Participants randomly assigned the 12-week programme lost significantly more weight than the brief intervention group at 3 months (adjusted difference −2·79 kg, 95% CI −3·44 to −2·13; p<0·0001) and 12 months (−1·61 kg, −2·84 to −0·38; p=0·0105), but there was no significant difference in weight loss between these groups at 24 months (−0·74 kg, −2·45 to 0·77; p=0·338).

Sensitivity analyses examining weight change with alternative assumptions about missing data gave similar results (appendix p 4). Research centre was not a predictor of weight change in any of the models. The intervention effect did not significantly differ by participant gender (p=0·48), educational attainment (p=0·79), or household income (p=0·64).

At 12-month follow-up, 57% of participants in the 52-week programme had lost 5% or more of weight, compared with 42% in the 12-week programme and 25% in the brief intervention (table 3). Participants in the behavioural programme were significantly more likely than the brief intervention group to lose 5% or more bodyweight. Participants in the 52-week programme were significantly more likely than those in the 12-week programme to lose 5% or more weight (table 3).

**Table 3:** Proportion of participants losing at least 5% and at least 10% baseline weight and relative risk for weight loss at 12 months and 24 months

	**Proportion of participants losing at least 5% baseline weight**			**Behavioural programme *vs* brief intervention**		**52-week programme *vs* 12-week programme**	
	Brief intervention	12-week programme	52-week programme	Relative risk (95% CI)	p value	Relative risk (95% CI)	p value
**≥5% weight loss**							
12 months	25% (2·97)	42% (2·15)	57% (2·16)	2·01 (1·51–2·67)	<0·0001	1·36 (1·14–1·62)	0·0005
24 months	22% (2·87)	27% (1·93)	39% (2·12)	1·47 (1·08–1·99)	0·0131	1·44 (1·16–1·78)	0·0009
**≥10% weight loss**							
12 months	9% (2·02)	15% (1·56)	30% (2·00)	2·40 (1·52–3·78)	0·0002	2·00 (1·53–2·62)	<0·0001
24 months	9% (1·93)	12% (1·43)	18% (1·69)	1·80 (1·11–2·93)	0·0182	1·49 (1·09–2·04)	0·0125

Data are % (SE) or relative risk (95% CI).

At 12 months and 24 months, participants in the 52-week programme had greater reductions in waist circumference and fat mass than participants in the 12-week programme or brief intervention group (table 4). At 12 months, participants in the 52-week programme had greater reductions in HbA_1c_ than those in the 12-week programme (adjusted difference −1·31 mmol/mol, 95% CI −2·47 to −0·15; p=0·0268) and brief intervention (−2·65 mmol/mol, −4·28 to −1·01; p=0·0015) and greater reductions in fasting plasma glucose concentration than those in the 12-week programme (−0·29 mmol/L, −0·58 to 0·00; p=0·0497) and brief intervention (−0·46 mmol/L, −0·88 to −0·03; p=0·0342). There were no significant differences between the 12-week programme and brief intervention for either HbA_1c_ or fasting glucose concentration. Changes over time in blood pressure, quality of life, triglycerides, HDL, LDL, and total cholesterol were small and no significant differences between groups were recorded. No participants reported adverse events related to the intervention.

**Table 4:** Changes from baseline in secondary outcomes and adjusted differences between each intervention at 3, 12, and 24 months

		**Change from baseline**			**52-week programme *vs* brief intervention**		**12-week programme *vs* brief intervention**		**52-week programme *vs* 12-week programme**	
	N	Brief intervention	12-week programme	52-week programme	Adjusted difference (95% CI)	p value	Adjusted difference (95% CI)	p value	Adjusted difference (95% CI)	p value
**3 months**										
Waist circumference (cm)	1266	−2·42 (0·49)	−4·66 (0·25)	−4·20 (0·25)	−1·78 (−2·71 to −0·84)	0·0002	−2·32 (−3·25 to −1·39)	<0·0001	0·54 (−0·08 to 1·17)	0·089
Fat mass (kg)	1236	−1·59 (0·30)	−3·95 (0·18)	−3·50 (0·14)	−1·85 (−2·43 to −1·27)	<0·0001	−2·31 (−2·90 to −1·72)	<0·0001	0·46 (0·04 to 0·88)	0·0324
Systolic blood pressure (mm Hg)	1263	−2·39 (1·01)	−5·50 (0·66)	−5·25 (0·61)	−2·82 (−5·11 to −0·53)	0·0158	−3·32 (−5·66 to −0·98)	0·0054	0·50 (−1·24 to 2·25)	0·571
Diastolic blood pressure (mm Hg)	1263	−2·59 (0·65)	−4·27 (0·41)	−3·64 (0·37)	−1·02 (−2·47 to 0·42)	0·164	−1·78 (−3·25 to −0·31)	0·0174	0·76 (−0·32 to 1·83)	0·166
Quality of life (EQ5D-3L tariff)	1209	−0·011 (0·016)	0·012 (0·009)	0·002 (0·009)	0·011 (−0·026 to 0·047)	0·562	0·023 (−0·013 to 0·060)	0·214	−0·013 (−0·036 to 0·011)	0·298
**12 months**										
Waist circumference (cm)	1266	−3·18 (0·64)	−5·15 (0·43)	−7·28 (0·45)	−4·05 (−5·54 to −2·56)	<0·0001	−2·12 (−3·59 to −0·65)	0·0048	−1·93 (−3·01 to −0·85)	0·0005
Fat mass (kg)	1236	−2·48 (0·55)	−3·71 (0·33)	−5·05 (0·35)	−2·84 (−3·91 to −1·77)	<0·0001	−1·40 (−2·47 to −0·33)	0·0102	−1·44 (−2·22 to −0·66)	0·0003
Systolic blood pressure (mm Hg)	1263	−2·77 (1·16)	−3·36 (0·73)	−3·74 (0·87)	−1·04 (−3·64 to 1·56)	0·433	−0·59 (−3·18 to 2·01)	0·657	−0·45 (−2·49 to 1·59)	0·664
Diastolic blood pressure (mm Hg)	1263	−1·64 (0·87)	−2·31 (0·43)	−2·71 (0·51)	−1·20 (−2·98 to 0·58)	0·186	−0·75 (−2·52 to 1·01)	0·403	−0·45 (−1·70 to 0·80)	0·483
Fasting glucose (mmol/L)	800	−0·11 (0·20)	−0·27 (0·10)	−0·54 (0·08)	−0·46 (−0·88 to −0·03)	0·0342	−0·17 (−0·59 to 0·26)	0·436	−0·29 (−0·58 to 0·00*)	0·0497
HbA_1c_ (mmol/mol)	835	0·15 (0·69)	−1·49 (0·37)	−2·77 (0·47)	−2·65 (−4·28 to −1·01)	0·0015	−1·34 (−2·96 to 0·29)	0·107	−1·31 (−2·47 to −0·15)	0·0268
HbA_1c_ (%)	835	0·01 (0·07)	−0·13 (0·03)	−0·24 (0·04)	−0·24 (−0·39 to −0·09)	0·0015	−0·12 (−0·27 to 0·03)	0·107	−0·12 (−0·23 to −0·01)	0·0268
Triglycerides (mmol/L)	837	−0·14 (0·07)	−0·23 (0·05)	−0·26 (0·03)	−0·09 (−0·25 to 0·07)	0·281	−0·06 (−0·22 to 0·10)	0·451	−0·03 (−0·14 to 0·09)	0·646
Cholesterol (mmol/L)	837	−0·31 (0·10)	−0·32 (0·05)	−0·36 (0·05)	−0·05 (−0·24 to 0·14)	0·625	−0·01 (−0·20 to 0·19)	0·938	−0·04 (−0·17 to 0·09)	0·556
LDL cholesterol (mmol/L)	830	0·01 (0·10)	0·02 (0·05)	0·02 (0·05)	0·00* (−0·19 to 0·19)	0·9996	0·00 (−0·19 to 0·19)	0·976	0·00 (−0·13 to 0·14)	0·964
HDL cholesterol (mmol/L)	837	−0·27 (0·04)	−0·24 (0·03)	−0·24 (0·03)	0·01 (−0·12 to 0·13)	0·917	0·03 (−0·10 to 0·15)	0·668	−0·02 (−0·11 to 0·07)	0·641
Quality of life (EQ5D-3L tariff)	1209	−0·014 (0·018)	0·009 (0·011)	−0·012 (0·011)	0·014 (−0·025 to 0·054)	0·476	0·029 (−0·011 to 0·069)	0·150	−0·015 (−0·044 to 0·014)	0·323
**24 months**										
Waist circumference (cm)	1266	−3·64 (0·72)	−4·36 (0·47)	−5·57 (0·45)	−1·98 (−3·56 to −0·41)	0·0137	−0·72 (−2·27 to 0·83)	0·365	−1·27 (−2·46 to −0·07)	0·0384
Fat mass (kg)	1236	−2·24 (0·62)	−2·40 (0·32)	−3·38 (0·38)	−1·36 (−2·64 to −0·08)	0·0375	−0·36 (−1·60 to 0·88)	0·572	−1·00 (−1·94 to −0·07)	0·0359
Systolic blood pressure (mm Hg)	1263	0·64 (1·13)	−0·85 (0·87)	−0·09 (0·78)	−0·52 (−3·07 to 2·02)	0·687	−1·22 (−3·75 to 1·31)	0·344	0·70 (−1·32 to 2·71)	0·497
Diastolic blood pressure (mm Hg)	1263	−0·83 (0·79)	−1·29 (0·50)	−1·11 (0·50)	−0·22 (−1·97 to 1·52)	0·803	−0·32 (−2·05 to 1·40)	0·713	0·10 (−1·13 to 1·34)	0·872
Quality of life (EQ5D-3L tariff)	1209	−0·005 (0·018)	−0·015 (0·012)	−0·018 (0·011)	−0·014 (−0·052 to 0·025)	0·486	−0·011 (−0·050 to 0·028)	0·587	−0·003 (−0·032 to 0·027)	0·843

Data are mean (SE). Analyses adjusted for baseline observation and centre. Mean weight change analyses use 20 imputed datasets. Treatment effects obtained from mixed-effects models with residuals structured as a first-order auto-regressive process stratified by treatment group. HBA_1c_=glycated haemoglobin A_1c_. EQ5D-3L=EuroQol 5-dimension 3-level. EQ-Vas=EuroQol Visual Analogue Scale.

* Rounded from <0·00.

At 3 months, 950 participants reported intervention use at 3 months. Seven (5%) of 132 participants in the brief intervention group had attended a commercial weight-management programme, compared with 259 (68%) of 382 participants in the 12-week programme and 300 (69%) of 436 participants in the 52-week programme (table 5). Only ten (1%) of 950 participants in all groups attended an NHS-led programme and three (<1%) used weight-loss medication. For participants referred to the behavioural programmes, the mean number of sessions attended was 8·4 (SD 4·2) in the 12-week programme and 28·2 (SD 14·8) in the 52-week programme. Full details of the economic evaluation are in the appendix (p 5). Briefly, intervention costs, including general practitioner referral time, are estimated at £18·50 (brief intervention), £60 (12-week programme), and £195 (52-week programme). There were no significant differences between groups in health-care resource use per participant over the 24-month follow-up period. The estimated incremental NHS cost per additional kg weight lost (expressed as £ per kg) was £91 per kg for the 12-week programme and £159 per kg for the 52-week programme. Analysis using a 1-year time horizon for consistency with other studies, reduced the ICER to £26 per kg for the 12-week programme and £75 per kg for the 52-week programme.

**Table 5:** Self-reported intervention use in the previous 3 months, recorded at 3, 12, and 24 months

	**3 months**			**12 months**			**24 months**		
	Brief intervention	12-week programme	52-week programme	Brief intervention	12-week programme	52-week programme	Brief intervention	12-week programme	52-week programme
Attendance questionnaire returned	132 (63%)	382 (72%)	436 (83%)	108 (51%)	321 (61%)	342 (65%)	115 (55%)	321 (61%)	330 (63%)
Attended one or more meeting of a commercial weight loss programme in past 3 months	7 (5%)	259 (68%)	300 (69%)	10 (9%)	60 (19%)	143 (42%)	14 (12%)	44 (14%)	57 (17%)
Attended nine or more meetings of a commercial weight loss programme in past 3 months	3 (2%)	199 (52%)	216 (50%)	8 (7%)	47 (15%)	100 (29%)	7 (6%)	25 (8%)	38 (12%)
Attended an NHS-led programme in past 3 months	1 (1%)	4 (1%)	5 (1%)	2 (2%)	3 (1%)	2 (1%)	4 (4%)	1 (<1%)	2 (1%)
Used weight-loss medication in past 3 months	0 (0)	1 (<1%)	2 (1%)	0 (0)	0 (0)	1 (<1%)	1 (1%)	0 (0)	1 (<1%)

Data are n or n (%).

The cost-effectiveness acceptability curve (appendix p 11) is based on weight loss at 2 years and includes all NHS costs incurred during that time. If decision makers are willing to pay at least £60 per kg, then the 12-week programme would be the preferred strategy. If decision makers are willing to pay £200 per kg, then the 52-week programme is preferable. If costs are restricted to intervention-only costs, the 52-week programme becomes the preferred strategy at £100 per kg.

Microsimulation modelling estimated that over 25 years after the baseline year, the 12-week programme was dominant in health economic terms (cost-saving and resulted in greater health benefits) compared with the brief intervention. The 52-week programme was cost-effective relative to the brief intervention (ICER £2493 per QALY) and the 12-week programme (£3804 per QALY).

By comparison with the brief intervention, the 12-week programme resulted in 623 fewer incident cases of disease, 643 additional QALYs, and a cost-saving of approximately £268 000 per 100 000 individuals. By comparison with the 12-week programme, the 52-week programme resulted in 1786 fewer incident cases of disease and generated 1282 additional QALYs, at a cost of approximately £4·9 million per 100 000 individuals (for further details see appendix p 17).

## Discussion

Adults in primary care who were overweight or obese and who were referred to an open-group behavioural weight-management programme lost more weight at 12-month follow-up than those who were given brief advice and self-help materials. People who were referred to this behavioural programme for 52 weeks lost more weight than those who were referred for 12 weeks. 57% of participants referred to the 52-week programme lost more than 5% weight, compared with 42% referred to the 12-week programme and 25% of those in the brief intervention group. 5% weight loss is often used as a cut-off for clinically significant weight loss, although even smaller weight losses are associated with improvements in markers of cardiovascular disease risk.^20^ All groups regained some of the weight lost and at 24 months the difference in weight loss between the 12-week programme and the brief intervention was no longer significant, whereas the weight loss in the 52-week programme was significantly higher than both other groups. Participants in the 52-week programme also had larger reductions in waist circumference, fat mass, fasting glucose concentration, and HbA_1c_ than participants in the 12-week programme and the brief intervention. When the impact of the 12-week programme was modelled over 25 years, it was cost-saving compared with the brief intervention. Although the 52-week programme was more expensive in the within-trial analysis, when the impact was modelled over 25 years, the 52-week programme resulted in the greatest gain in QALYs and the greatest reduction in disease incidence. By standards set by the National Institute of Health and Care Excellence, the 52-week programme is cost-effective compared with the brief intervention and the 12-week programme. This assessment of cost-effectiveness does not include potential further savings in social care and indirect health-care costs. It also uses a flat-rate cost for all participants who took up an intervention, whereas, in some payment models, full costs might not be incurred if people did not complete the course, thus potentially overestimating intervention costs.

A strength of this trial is the large patient group that is broadly generalisable to the UK population. In our previous trial,^13^ participants with a BMI of 27–35 kg/m^2^ were identified during routine consultations and recruited to the trial. This trial was more inclusive (BMI 28–68 kg/m^2^) and participants were recruited by letter based on their weight records. In the 2011 trial, the mean BMI was 31·4 kg/m^2^ whereas in the present study participants had a mean BMI of 34·5 kg/m^2^, which is more comparable to the population typically referred to open-group behavioural programmes in NHS referral schemes.^21^ Invitation of all eligible patients by mail resulted in a higher proportion of men recruited than seen in routine referral schemes,^13^, ^21^ although this percentage is still lower than the proportion of men in the UK population.^22^ More than half of participating practices were from areas with an index of multiple deprivation that is higher (more deprived) than the national median and participants were drawn from a wide range of socioeconomic groups.^23^ Most participants were white, with the proportion enrolled reflecting the ethnic composition of the UK.^22^ Intervention effects did not vary by gender or socioeconomic status. This finding is striking given often repeated views that these interventions are not appropriate for men and concerns that individual behavioural interventions exacerbate socioeconomic inequalities in health.^24^, ^25^ Taken together, the findings suggest that the intervention effects reported here might be generalisable to the UK adult population. Although uptake of the programmes by more deprived populations is somewhat lower than in less deprived areas,^23^ more targeted schemes, as have been implemented elsewhere,^14^ might reduce health inequalities.

Loss to follow-up at 1 year in our study was slightly below average for weight-loss trials,^26^ and there was little attrition between 1 and 2 years. Three different intention-to-treat analyses were done that each make different assumptions about missing data, as well as two sensitivity analyses. The consistency of effects shows the robustness of our findings. The pragmatic nature of the trial meant that participants in all groups were free to use other weight-loss methods during the trial. This reflects how these interventions are routinely delivered and allows direct translation of these findings to clinical practice. Only very few participants in each group used other NHS interventions or weight-loss medications, and only a small proportion of participants who were assigned to the brief intervention group went on to use a commercial weight-loss programme. A strength of this trial is the 2-year follow-up of participants, which gives important information on weight trajectories after participation in the programme ends. The modelled cost-effectiveness over 25 years is based on assumptions about weight trajectories beyond 2 years; however, we have been conservative in these assumptions, assuming all weight lost is regained within 5 years. The within-trial ICERs are strongly affected by non-significant differences between groups in health-care costs (highest in the 52-week programme and lowest in the 12-week programme). However, sensitivity analyses (appendix p 9) provide information with which to assess the strength of these findings. Fasting status when blood was taken was self-reported and could be susceptible to reporting bias or recall bias. Reliance on participants confirming that the blood sample was taken in a fasting state is a universal concern in any free-living study and findings related to glucose were congruent with findings for HbA_1c_, which is not affected by fasting status. Attendance at sessions was self-reported and could be susceptible to reporting, or recall bias, or both. However, any bias in the attendance data does not affect the prespecified outcomes, which were done on an intention-to-treat basis.

The weight losses seen in this study are consistent with previous trials^13^, ^14^, ^27^ of referrals to 12-week and 52-week commercial, open-group behavioural weight-management programmes, suggesting that our findings are robust. Estimates of the mean incremental cost per additional kg of bodyweight lost for either duration of treatment are in the range of other behavioural programmes (appendix p 12). The reductions in fasting glucose concentration and HbA_1c_ were not seen in our previous trial^13^ of the 52-week programme, perhaps because the lower baseline BMI and stricter inclusion criteria in that study meant that participants had lower baseline values than in this study. Almost half of participants in the present trial had elevated fasting glucose concentration, HbA_1c_, or both at baseline. Reductions seen in participants in the 52-week programme group at 12 months (−0·5 mmol/L fasting glucose and −2·8 mmol/mol HbA_1c_ [–0·24% HbA_1c_]) are larger than those seen at the same timepoint in the intensive lifestyle intervention arm of the Diabetes Prevention Programme (DPP; approximately −0·4 mmol/L fasting glucose and −1 mmol/mol HbA_1c_^28^), in which participants were similar to those in the present study in baseline BMI, HbA_1c_, and fasting glucose concentration, and had similar weight loss at 12 months, but achieved at a fraction of the cost.^29^ Notwithstanding gradual weight regain and increase in associated risk factors observed over 15 years of follow-up, DPP achieved a 27% reduction in the cumulative incidence of diabetes in the lifestyle intervention relative to the control group.^30^ However, the impact of these more scalable interventions on diabetes incidence will depend on whether longer term weight trajectories are similar.

The weight-loss programme we assessed is widely available and participants in the brief intervention group were neither encouraged nor discouraged from attending other weight-loss programmes. Only one in 20 chose to do so, compared with 14 in 20 of those referred to these programmes. In the 12-week group, 19% of participants were still attending the programme 12 months later at their own cost, compared with 9% of those in the brief intervention. This finding suggests a legacy effect of the initial referral. However, this proportion of participants is lower than that of the 52-week group (42% of participants were still attending at 12 months). Because the median household income was about £30 000, many people would probably have been able to afford the programme (approximately £5 per week) suggesting that the act of referral itself might have been the source of motivation to attend, although it is impossible to exclude the possibility that lower attendance in other groups was related to the cost. The importance of the referral is supported by qualitative data that found the general practioner referral is perceived as an implicit recommendation of the programme and the allocation of NHS resources to enable their attendance increases motivation to attend.^31^ These findings are supported by another trial^32^ in people who were obese attending a general practitioner consultation unrelated to their weight, in which 77% who agreed to participate in the trial and were offered a referral to a weight-management programme accepted it and 40% attended the programme.

The absolute weight loss in participants who had the brief intervention also warrants consideration. Participants given 5 min of non-tailored advice and a self-help booklet lost more than 3 kg at 12 months and more than 2 kg at 24 months. Weight change in this group is higher than the average weight loss observed in a meta-analysis^3^ for interventions led by generalist primary care teams, and higher than the average weight loss seen in a systematic review^33^ of self-help interventions. Given the small cost of this intervention (self-help booklet and three short appointments that could be given by a nurse or health-care assistant), observational data support the implementation of this intervention as a minimal standard in primary care. However, this finding highlights the importance of including a control group when evaluating the effects of weight-loss interventions. Findings from a review^34^ have shown that control groups given minimal interventions will generally lose weight over the course of a trial. The absolute weight loss of control groups was considerably heterogeneous in this review,^34^ which might reflect differences in study populations and trial design. Our trial illustrates this disparity between trials, showing 3·3 kg weight loss in the brief intervention control group, when an almost identical brief intervention resulted in less than 1 kg weight loss when used as a control group in a different population and context.^35^

This trial shows that referral to this commercial open-group behavioural weight-loss programme increases weight loss relative to a brief intervention in primary care. Increasing the duration of the programme from 12 weeks to 52 weeks increases weight loss and improvements in other markers of diabetes and cardiovascular risk, most notably HBA_1c_ and fasting glucose concentration. Economic evaluation of this trial found that although the 52-week programme requires greater initial investment, the programme is likely to be cost-effective in the long-term and health-care providers should consider a move towards extended referral schemes.

**This online publication has been corrected. The corrected version first appeared at thelancet.com on May 17, 2017**
