# Department of Error

**DOI:** 10.1016/S0140-6736(17)31257-6

**Published:** 2017-06-03

*Ahern AL, Wheeler GM, Aveyard P, et al. Extended and standard duration weight-loss programme referrals for adults in primary care (WRAP): a randomised controlled trial.* Lancet *2017; **389:** 2214–25*—In table 5 of this Article, the percentage of participants in the brief intervention group who reported attending nine or more meetings should have been 7%. This change has been made to the online version as of May 17, 2017, and the printed Article is correct.

