# Supplementary material for: Extended and standard duration weight-loss programme referrals for adults in primary care (WRAP): a randomised controlled trial
Source: Lancet. 2017 Jun 3;389(10085):2214–25. doi: 10.1016/S0140-6736(17)30647-5 (PMC5459752; doi:10.1016/S0140-6736(17)30647-5)
Supplement: Supplementary appendix [file mmc1.pdf]

THE LANCET

Supplementary appendix

This appendix formed part of the original submission and has been peer reviewed. We post it as supplied by the authors.

Supplement to: Ahern AL, Wheeler GM, Aveyard P, et al. Extended and standard duration weight-loss programme referrals for adults in primary care (WRAP): a randomised controlled trial. *Lancet* 2017; published online May 3. http://dx.doi.org/10.1016/S0140-6736(17)30647-5.

Extended and standard duration weight loss referrals for adults in primary care (WRAP): a pragmatic randomised controlled trial.

Web Appendix

1. **Analysis of Blood Samples**
2. **Sensitivity Analyses of Clinical Outcomes**
3. **Within Trial Cost-Effectiveness Analysis**
4. **Long Term Cost-Effectiveness Modelling**
5. **References**

1. Analysis of Blood Samples

All methods were externally assessed regularly, using NEQAS or RIQAS external quality assessment schemes.

Total cholesterol: Cholesterol is released from its esters using cholesterol esterase; this and endogenous free cholesterol are oxidised using cholesterol oxidase which produces hydrogen peroxide. The hydrogen peroxide is quantitated by enzymic oxidation of N,N diethylamine hydrochloride/4-aminoantipyrine to form a coloured chromophore absorbing at 540 nm. Absorbance is measured at 452, 540 and 700 nm to provide specificity. Between-batch precision of this assay was between 1% and 3%.

HDL cholesterol: HDL cholesterol was measured on the Siemens Dimension Xpand. HDL cholesterol is quantitated by reactions analogous to those used for total cholesterol, using PEG-modified cholesterol esterase and cholesterol oxidase. This follows pretreatment of the serum with dextran sulphate and magnesium sulphate which reacts with chylomicrons, LDL and VLDL to prevent them from interfering. The cholesterol oxidase reaction produces hydrogen peroxide which is quantitated colorimetrically as for total cholesterol, by generating a coloured dye using peroxidase. Between –batch precision of this assay was between 3% and 8%.

Triglycerides: The sample is first hydrolysed with lipoprotein lipase to release glycerol from fatty acids. Glycerol is then phosphorylated using glycerol kinase and the glycerol phosphate thus produced is oxidised by glycerol-3-phosphate oxidase, generating hydrogen peroxide. Hydrogen peroxide is quantitated colorimetrically using peroxidase, aminoantipyrine and 4-chlorophenicol, producing a quinoneimine chromophore which absorbs at 510 nm. Absorbance is also measured at 700 nm to allow for turbidity. Between-batch precision of this assay was between 2% and 5%.

Glucose: Glucose is phosphorylated using hexokinase and then oxidised using glucose-6-phosphate dehydrogenase, generating NADH from NAD. The NADH produced is measured spectrophotometrically at the endpoint of the reaction from absorbance at 340 nm and 383 nm. Between-batch precision of this assay was between 1% and 5%.

2. Sensitivity Analyses of the Primary Outcome

2.1 Assumptions about missing data

Table A1 shows differences in mean weight change at each time point using different assumptions about missing data: Baseline Observation Carried Forward, Last Observation Carried Forward, and Completers Only. These assumptions do not substantially change the direction or magnitude of effects for these analyses.

2.2 Inclusion of self-reported data

Incorporating self-reported weight data from 80 participants who did not attend the 12 month assessment increased 12 month retention to 71% but did not significantly affect the direction or magnitude of intervention effects. Mean weight change at 12 months was -3·35 kg (0·67) in brief intervention, -4·76 kg (0·33) in the 12-week programme, and -6·69 kg (0·39) in the 52-week programme, with an adjusted difference of -2·55 kg (-3·69, -1·40) between combined behavioural programmes and brief intervention, and an adjusted difference of -1·98 kg (-2·87, -1·09) between the 12-week and 52-week programmes.

2.3. Restricting the assessment window

Restricting the primary analyses to the 769 participants who completed the 12 month assessment visit within -1/+2 months of the due date did not change the direction or magnitude of effects. Mean weight change was -3·33kg (0·66) in brief intervention, -5·35kg (0·41) in the 12-week programme, -7·66 kg (0·46) in the 52-week programme, with an adjusted difference of -3·27 kg (-4·82, -1·71) between combined behavioural programmes and brief intervention, and -2·39 kg (-3·58, -1·21) between the 12-week and 52-week programmes.

Table A1: Changes in weight from baseline (mean, SE) at 3, 12, and 24 months by intervention group using different assumptions about missing data

		Intervention			Test 1 (One-sided) Adjusted Difference (95%CI)		Test 2 (Two-sided) Adjusted Difference (95%CI)	
	N	Brief Intervention	12-week Programme	52-week Programme	Behavioural Programmes vs Brief Intervention	p-value	52-week Programme vs 12-week Programme	p-value
**Weight change (kg) at 3 months**								
MAR*	1267	-2·04 (0·30)	-4·84 (0·19)	-4·62 (0·17)	-2·67 (-3·28, -2·07)	<0·0001	0·22 (-0·26, 0·69)	0·371
BOCF	1267	-1·47 (0·22)	-3·75 (0·18)	-4·04 (0·17)	-2·43 (-2·99, -1·86)	<0·0001	-0·32 (-0·78, 0·14)	0·178
LOCF	1267	-1·47 (0·22)	-3·75 (0·18)	-4·04 (0·17)	-2·43 (-2·99, -1·86)	<0·0001	-0·32 (-0·78, 0·14)	0·178
Completers	1004	-2·15 (0·31)	-4·89 (0·20)	-4·68 (0·18)	-2·68 (-3·35, -2·01)	<0·0001	0·19 (-0·32, 0·70)	0·456
**Weight change (kg) at 12 months**								
MAR*	1267	-3·26 (0·68)	-4·75 (0·35)	-6·76 (0·42)	-2·71 (-3·86, -1·55)	<0·0001	-2·14 (-3·05, -1·22)	<0·0001
BOCF	1267	-1·93 (0·38)	-3·27 (0·27)	-5·12 (0·34)	-2·27 (-3·28, -1·26)	<0·0001	-1·88 (-2·70, -1·06)	<0·0001
LOCF	1267	-2·30 (0·40)	-3·94 (0·28)	-5·96 (0·33)	-2·65 (-3·66, -1·63)	<0·0001	-2·06 (-2·88, -1·24)	<0·0001
Completers	823	-3·28 (0·62)	-5·10 (0·39)	-7·52 (0·44)	-3·12 (-4·59, -1·65)	<0·0001	-2·53 (-3·66, -1·39)	<0·0001
**Weight change (kg) at 24 months**								
MAR*	1267	-2·30 (0·73)	-3·00 (0·37)	-4·29 (0·44)	-1·44 (-2·87, -0·00)	0·0247	-1·32 (-2·46, -0·18)	0·0231
BOCF	1267	-1·41 (0·45)	-2·06 (0·27)	-3·17 (0·33)	-1·21 (-2·23, -0·20)	0·0097	-1·13 (-1·96, -0·31)	0·0073
LOCF	1267	-1·85 (0·47)	-2·73 (0·28)	-4·16 (0·34)	-1·59 (-2·65, -0·54)	0·0016	-1·47 (-2·32, -0·61)	0·0008
Completers	856	-2·23 (0·70)	-3·07 (0·39)	-4·55 (0·46)	-1·66 (-3·16, -0·16)	0·0148	-1·52 (-2·70, -0·34)	0·0113

*MAR = Missing at random analysis; BOCF= baseline observation carried forward; LOCF = last observation carried forward
Adjusted differences are shown between combined open-group behavioural programme groups (Behavioural Programmes) and Brief Intervention (Test 1) and between the 12-week programme and the 52-week programme (Test2)
Analyses are adjusted for baseline observation and centre

3. Within-Trial Cost-Effectiveness Analysis

3.1 Economic Analysis

We performed within-trial cost–effectiveness analysis with a 2-year time horizon, examining incremental cost per additional kg weight loss. Costs were assessed from an NHS & personal social services perspective for the financial year 2014-15, and costs incurred in the second year were discounted at 3·5%.[1]

Intervention costs: The cost of the British Heart Foundation booklet given to the brief intervention participants was £2·50, based on previous models.[2] In costing the open-group behavioural programme (Weight Watchers®), we applied the price which would be charged to the NHS, as opposed to estimating the costs to individuals attending from the community. We assumed that if the participant attended at least one session, the NHS would be charged £48·50 (12-week programme) or £190 (52-week programme). If they did not attend, there was no charge. In practice, full costs may not be paid where participants did not attend further meetings, so this may overestimate intervention costs. At randomisation, all participants received a brief introduction to their intervention. If rolled out, this was estimated to add approximately 5 minutes to a standard GP consultation, the cost of which (£16)[3] was added to all participants.

Non-intervention NHS costs were estimated from health resource use questionnaires, which were completed by participants at baseline, 3, 12, and 24 months. Associated unit costs were obtained from published reference sources[3-5] (available on request). Health resource use included number of visits to GP and other community health care workers; out-patient appointments; accident and emergency; inpatient stays and prescribed medications. All resource use questionnaires were framed within a three-month recall period. To ascertain the full NHS costs incurred over the 24-month follow-up period, we applied area-under-the-curve methods.[6]

To account for missing data, our multiple imputation model included predictors of total costs and outcomes (health resource use costs and weight at 0, 3, 12, and 24 months), treatment arm, centre, age, sex, and centre. Imputation took place in 20 cycles.[7] With complete cost and outcomes at all four time points, we used area-under-the-curve analysis to generate cost and weight estimates for all participants. This enabled paired cost and outcome data for the entire study population (n=1267).

The primary economic analysis compared costs to kg weight loss. We conducted bivariate regression analyses of cost and weight, adjusting for age, sex, and centre. Regression coefficients were used to estimate the incremental cost-effectiveness ratio (ICER), that is, the difference in mean costs (£) divided by the difference in mean weight loss (kg) between groups. As there are considerable uncertainties involved in estimating the ICER, presenting only a single point estimate of an ICER is unlikely to provide adequate information for decision-making. To capture the uncertainty around these estimates, we used nonparametric bootstrapping, plotting 1000 paired cost and effect (for the 12-week programme versus brief intervention, and the 52-week programme versus brief intervention) on a cost-effectiveness plane, where incremental cost is plotted on the *y* axis and incremental effect is plotted on the *x* axis. Finally, we produced cost-effectiveness acceptability curves (CEACs) to depict the probability that each intervention strategy is cost-effective at varying incremental cost per additional kg lost thresholds.[8]

Our primary economic analysis assessed incremental cost per additional kg weight loss over 2 years (expressed as £/kg). To assess the robustness of the analysis to changes in key input values and assumptions, we considered the following sensitivity analyses:

1. Incremental cost per additional kg weight loss using complete case dataset
2. Incremental cost per additional kg weight loss excluding non-intervention health resource use costs

To be more comparable with other studies with shorter time horizons, we also calculated incremental cost per kg at 1 year, with and without non-intervention health resource use costs.

3.2 Results

3.2.1. Data availability

3731 out of 5068 (73·6%) health resource use questionnaires were returned. In order to estimate total cost effectiveness, we required complete cost and outcome data at all four time points. Complete cost per kg lost data was available for 617 (46·7%) participants.

5.2.2. Cost analysis

All brief intervention participants received the BHF booklet. This cost £18·50 per person including GP time. 49 participants (9·3%) in the 12-week programme and 31 (5·9%) in the 52-week programme did not attend any Weight Watchers® sessions, so only the £16 GP contact cost was applied for these participants. The mean number of sessions attended was 8·4 (SD 4·2) in the 12-week programme and 28·2 (SD 14·8) in the 52-week programme. The Weight Watchers® intervention was estimated to cost £60 per participant in the 12-week programme and £195 in the 52-week programme.

Health care resource use per participant was broadly similar between arms at baseline and throughout follow-up (Table A2). Over the two years examined, the cost of funding the open-group behavioural programme was not offset by lower costs in terms of GP and other community contacts, secondary care, or medications. The 12-week programme participants reported lowest health resource use costs in all categories apart from medications (where brief intervention reported the lowest costs) and the 52-week programme costs were highest in most resource use categories. However, between-group differences in resource use were not statistically significant. Overall, NHS resource use costs were lowest in brief intervention (£1668) and highest in the 52-week programme (£1799), but these differences were also not statistically significant.

3.2.3 Incremental cost per additional kg lost (£/kg): base case analysis

When the cost of each intervention was added to health care costs, total NHS costs were highest in the 52-week programme group. When compared with Brief Intervention, the incremental cost of the open-group behavioural weight-loss programmes was £51·25 (CI -321 to 424, p=0·27) for the 12-week programme and £309·50 (CI -63 to 682, p=0·10) for the 52-week programme. Incremental weight loss at 2 years was non-significant in the 12-week programme (-0·56kg, CI -1·68 to 0·55, p=0·32), and statistically significant in the 52-week programme (-1·94kg, CI -3·05 to 0·83, p=0·001), producing an ICER of £91/kg lost for the 12 week programme and £159/kg lost for the 52-week programme, both compared to brief intervention.

A summary of costs and effects at base-case and sensitivity analyses, along with cost-effectiveness ratios, is provided in Table A3. Limiting the costs to intervention only reduced the ICER to £73/kg for the 12-week programme and £91/kg for the 52-week programme. Analysis of cost/kg using a 1-year time horizon (Table A4) reduced the ICER to £26/kg and £75/kg for the 12-week and 52-week programmes respectively (£28/kg and £50/kg respectively for intervention only costs). Table A5 presents a summary of the cost-effectiveness results from broadly related behavioural intervention studies that included weight loss as one desired outcome. The majority of these studies provide cost/kg with a 1-year time horizon

3.2.4 Analysis of uncertainty

Figure A1 shows the spread of bootstrapped paired estimates on a cost effectiveness plane. In the 52-week programme arm, the overwhelming majority of cost-effect pairs (96% of samples) are located in the North East (NE) quadrant, where the 52-week programme costs more and leads to more weight loss, compared to brief intervention. For the 12-week programme, cost-effect pairs are distributed more widely, with only 51% in the NE quadrant and 34% in the dominant South East quadrant (where the 12-week programme costs *less* and loses more weight compared to brief intervention).

The cost-effectiveness acceptability curve (CEAC, Figure A2) shows the probability that each weight loss strategy is cost-effective for a range of values for the cost-effectiveness threshold. If the threshold was between £0 and about £60 for a weight loss of 1 kg, then the Brief Intervention model has the highest probability of being cost-effective. As the threshold increases, implying that more value is placed on weight loss (i.e. policymakers are willing to pay more per kg lost), then the open-group behavioural programmes become more cost-effective. At a value of between approximately £60 and £200 pounds for each additional kg lost the 12-week programme has the highest probability of being cost-effective. If the threshold exceeds £200 per additional kg lost, then the 52-week programme has the highest probability of being cost-effective.

Because differences in health resource use were not significantly different between groups, we also ran the CEAC using intervention-only costs (available on request.) Under this model, the 12-week programme has the highest probability of being cost-effective if policy makers are willing to pay between £70 and £100 per additional kg lost, and the 52-week programme is most likely to be cost-effective for values of over £100 per additional kg lost.

Table A2 Resource costs (complete case data)

	52-week Programme	12-week Programme	Brief Intervention
Cost Item	Mean cost/level of resource use	Mean cost/level of resource use	Mean cost/level of resource use
Intervention Costs	£194·84	£60·00	£18·50
Primary care	£342·08	£310·34	£321·39
Inpatient stays	£530·31	£449·29	£515·38
Outpatient & A&E	£459·89	£443·59	£466·29
Community care	£139·47	£116·28	£125·04
Medications	£341·06	£348·63	£299·12
Total NHS costs	£1,812·80	£1,668·13	£1,727·22
NHS + Intervention Costs	£2,007·64	£1,728·13	£1745·72

Primary care included GP surgery/home/phone and practice nurse surgery/phone
Inpatient stays were costed as per episode (does not incorporate length of stay)
Outpatient & A&E included consultant-led outpatient appointments, day hospitals, A&E and day case operations
Community care included contact with Physio, OT, psychologist, counsellor, dietician, podiatrist services, and other less common attendances
Medications focused on routine prescriptions and excluded prescribed equipment, emollients and topical medications

Table A3 Cost per additional kg lost at 2 years (compared to Brief Intervention)

Model		Incremental cost	p	CI	kg lost	p	CI	ICER
**Primary Cost/kg analysis** All data, all NHS costs (n=1267)	12-week Programme	£51·25	0·787	-321, 423	-0·564	0·318	-1·68, 0·54	£90·86
	52-week Programme	£309·49	0·103	-63, 682	-1·940	0·001	-3·06, -0·83	£159·46
**Sensitivity analysis 1** **Complete case data** (n=617)	12-week Programme	£60·96	0·847	-557, 677	-0·585	0·568	-2·59, 1·4	£104·26
	52-week Programme	£332·75	0·288	-281, 947	-3·011	0·003	-5·01,-1·02	£110·49
**Sensitivity analysis 2** **Intervention costs only** (n=1267)	12-week Programme	£41·43	0·000	37, 46	-0·564	0·318	-1·68, 0·54	£73·46
	52-week Programme	£176·28	0·000	171, 181	-1·940	0·001	-3·06, -0·83	£90·87

Table A4 Cost per additional kg lost at 1 year (compared to Brief Intervention)

Model		Incremental cost	p	CI	kg lost	p	CI	ICER
**Cost/kg at 1 year** **Total NHS costs**(n=1267)	12-week Programme	£38·88	0·71	-166, 244	-1·48	0·01	-2·58, -0·39	£26·22
	52-week Programme	£261·27	0·01	57, 466	-3·49	0·00	-4·59, -2·40	£74·78
**Cost/kg at 1 year** **Intervention costs only** (n=1267)	12-week Programme	£41·43	0·00	37, 46	-1·48	0·01	-2·58, -0·39	£27·94
	52-week Programme	£176·28	0·00	172, 182	-3·49	0·00	-4·59, -2·40	£50·46

Figure A1 Cost effectiveness plane – Incremental cost per additional kg lost (2-year time horizon)

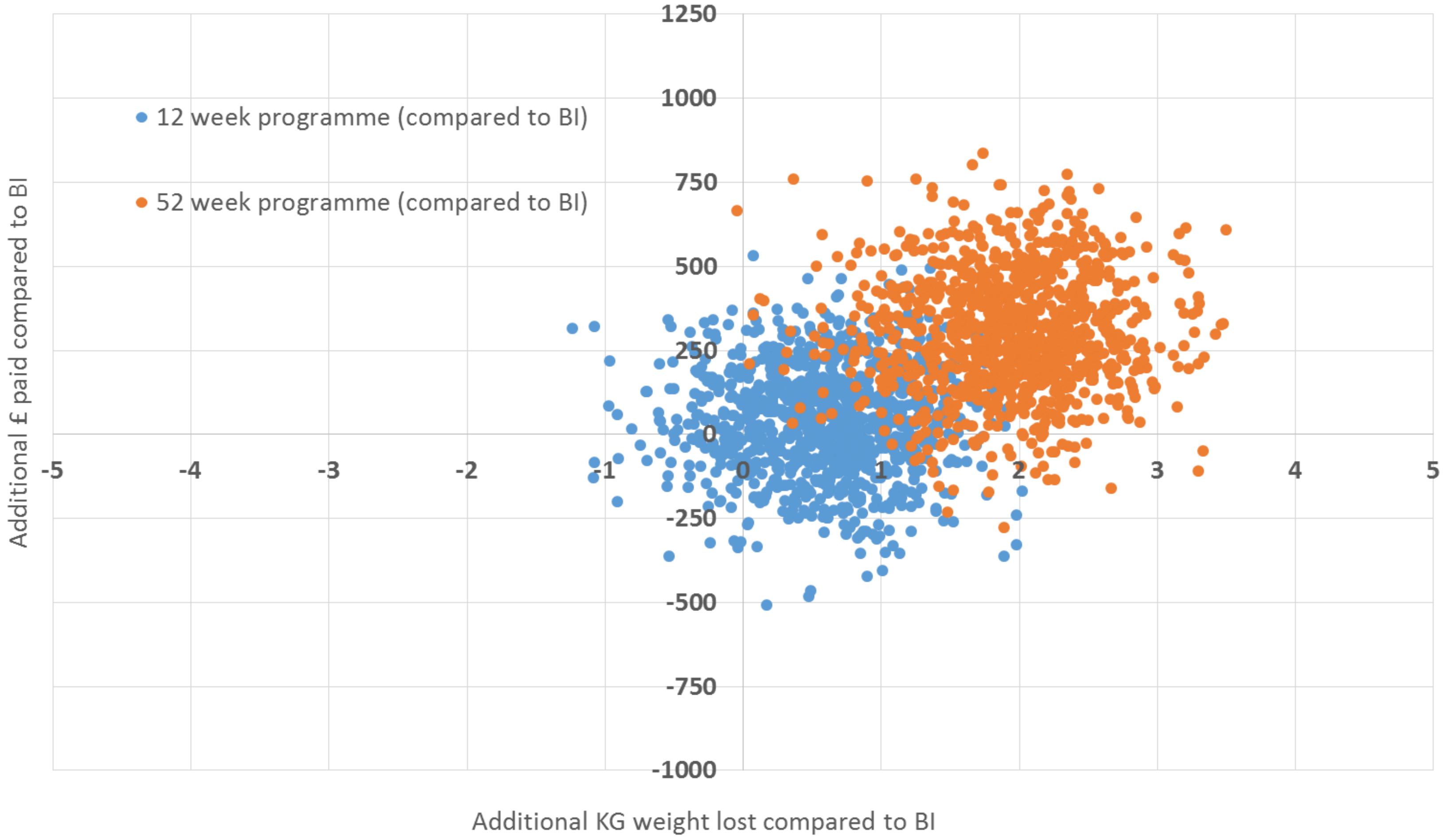

Figure A2 Cost Effectiveness Acceptability Curve (2-year time horizon)

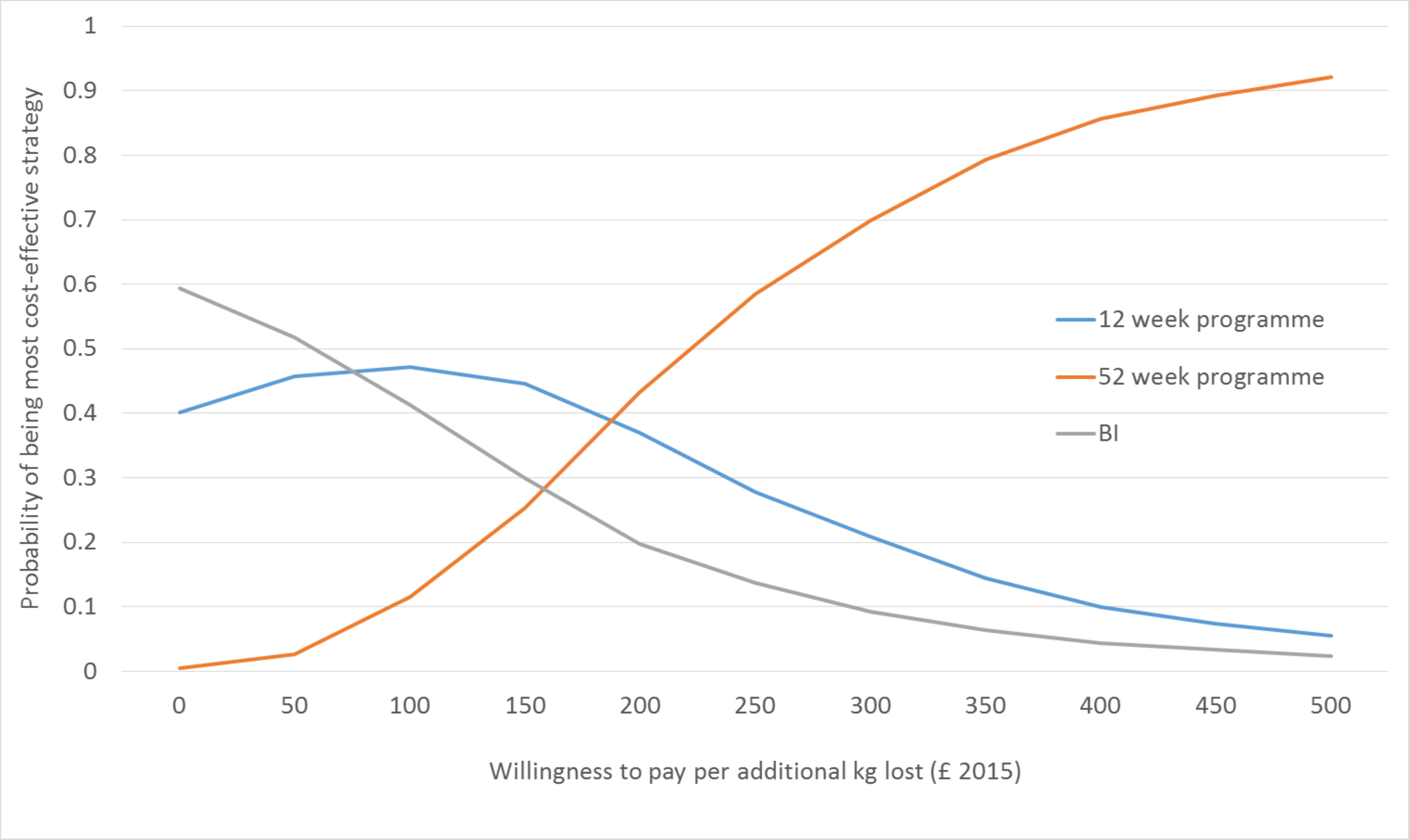

Table A5: Previous within-trial economic evaluations of weight management-related studies, using cost per kg as outcome

Author, year	Intervention	Time horizon	Cost per kg lost
Jebb 2011[9] / Fuller 2013[10]	Commercial weight loss programme	12 months	*UK: £ 55 (US$ 90)*<br>*Germany: US$ 180*<br>*Australia: US$ 122*<br>*(average cost-effectiveness)*
	Standard care (per national guidelines)		*UK: £92 (US$151)*<br>*Germany: US$133*<br>*Australia: US$138*<br>*(average cost-effectiveness)*
Tsai 2013[11]	Brief lifestyle counselling vs Usual care	24 months	$ 33 per kg-year§<br>(currency year not given)
	Enhanced brief lifestyle counselling vs Usual care		$ 201 per kg-year
	Enhanced brief lifestyle counselling vs Brief lifestyle counselling		$ 277 per kg-year
Disease Prevention Program (US)[12] ¥	Intensive group behavioural lifestyle modification		$ 159 per kg-year§<br>(2010 US dollars)
Gustafson 2009[13]	Intensive group behavioural lifestyle modification for low income women vs Do nothing	5 months	$ 55 per kg<br>$ 132 per kg-year§<br>(2007 US dollars)
Jakicic 2012[14]	Standard behavioural weight loss intervention (SBWI) vs STEP	18 months	$ 97 per kg (payer perspective)<br>$ 409 per kg (societal perspective)
	Stepped care weight loss intervention (STEP) vs "Status quo"		$ 58 per kg (payer perspective)<br>$ 127 per kg (societal perspective)
Krukowski 2011[15]	Group internet based weight loss intervention vs an identical intervention conducted in-person	6 months	The in person modality cost $88·31 per kg lost compared to the internet modality which cost $67.74 per kg lost

Hersey 2012[16]	Multicomponent behavioural weight loss programmes (MBWLP) (written materials and basic Web access (RCT1), plus an interactive Web site (RCT2))	12 months	$ 30-40 per 1 % weight loss* (2007 US$)<br>(average cost-effectiveness)
	MBWLP delivered by internet and telephone and email support (RCT3)		$ 70 per 1 % weight loss*<br>(average cost-effectiveness)
	RCT3 vs RCT1-RCT2		$ 200 per 1% weight loss*<br>(incremental cost-effectiveness)
Little 2016[17]	Web-based intervention and face-to-face nurse support vs. control (i.e. evidence-based dietetic advice & nurse follow-up)	12 months	£ 18 per kg lost
	Web-based intervention and remote nurse support vs control		£ -25 per kg lost

Note: *1% weight loss corresponds roughly to 1kg weight loss in the present study, given the mean initial weight of participants of about 95kg.

¥ The cost per kg for the Diabetes Prevention Program in the US[12] were estimated by Tsai et al. (2013). [11]

§ The cost per kilogram-year is defined as the cost of losing 1 kg of weight for one year.[11]

4. Long Term Cost-Effectiveness Modelling

4.1 Methods

A microsimulation model was used to simulate a virtual cohort for 25 years into the future. Using the framework of the Foresight: Tackling Obesities microsimulation[18,19], we projected the population's disease incidence, healthcare costs, quality adjusted life-years (QALY) and incremental cost effectiveness ratios (ICERs) in the years following baseline, in response to changes in BMI for three scenarios up to 2039 (25 years after WRAP baseline).

In the start year of the simulation (2014), the distribution of age, sex, and BMI of the whole WRAP study was used as the starting population. The model simulated the health experience of one individual at a time from the start year of the model simulation until 2039 (Figure A3). Firstly, the individual was randomly selected from the starting population and initialised with a BMI and a set of diseases based on the current health statistics (Table A6). Each year an individual's BMI was updated based on the specific WRAP intervention being modelled.

For the first two years, the mean change in BMI (using participants MAR weight at the time point and height measured at baseline) observed in one of the WRAP trial arms (brief intervention, 12-week programme, 52-week programme) was applied to the modelled population, assuming a one-off intervention. It was further assumed that weight was progressively regained to baseline values after 5 years from randomisation. Beyond this period, the BMI of simulated individuals was assumed to follow national trends determined by projecting longitudinal cross-sectional Health Survey for England BMI data.[20]

Based on this BMI change each year the disease status of the individual was updated. In the model, an individual may acquire one or more diseases based on their risk factor level, age, and sex. Any new incident cases of disease were recorded in each simulation year up until 2039. A number of diseases were modelled: coronary heart disease, stroke, diabetes, knee osteoarthritis, as well as BMI-related cancers, including breast, colorectal, endometrial, oesophageal, ovarian, pancreatic, and renal cancer. Each year an individual may die from one of these diseases or from another cause. If the individual died at the end of the year, they were not included in the model in subsequent years. If the individual was still alive at the end of the year the individual was included in the following year until death. Each year involved updating an individual's BMI (based on the intervention), disease, and mortality status. For each intervention the health experience of 20 million individuals were simulated.

Over the 25-year modelling horizon, disease incidence, associated healthcare costs, and QALYs were compared between scenarios. For a given comparison of A vs B, negative ICERs indicate that intervention A is either dominant in terms of cost-effectiveness ($Cost_A$ – $Cost_B$<0; $QALY_A$ – $QALY_B$>0) compared to B, or dominated by B ($Cost_A$ – $Cost_B$>0; $QALY_A$ – $QALY_B$<0). In such cases, absolute values for ICERs are not reported and the dominant or dominated intervention identified.

The microsimulation model does not currently produce estimates of uncertainty based on input data statistics (sensitivity analysis). However, the model does produce uncertainty arising from the Monte Carlo process, representing the accuracy of the microsimulation itself.

Figure A3 - Model Structure for an Individual simulated through their lifetime

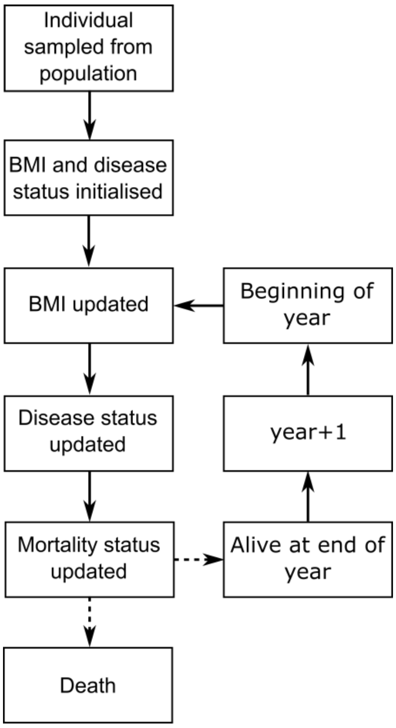

Table A6 – Source of disease data inputs

Disease	*Incidence*	*Prevalence*	*Mortality*	*Survival*	*Relative Risk*	*Utility*	*Direct costs*
CHD	*Smolina et al 2012. Corrected data on incidence and mortality in 2013* [21]	*BHF, Cardiovascular Disease Statistics 2014* [22]	*ONS, Deaths Registrations Summary Statistics, England and Wales, 2014* [23]	*Computed from prevalence and mortality*	*World Obesity Federation (DYNAMO project)* [24]	*Laires et al. 2015* [25]	*NHS 2012-13 programme budgeting data* [26]
Stroke	*BHF, stroke statistics 2009* [27]	*BHF, Cardiovascular Disease Statistics 2014* [22]	*ONS, Deaths Registrations Summary Statistics, England and Wales, 2014* [23]	*Computed from prevalence and mortality*	*World Obesity Federation (DYNAMO project)* [24]	*Rivero-Arias et al. 2010 -*[28]	*NHS 2012-13 programme budgeting data* [26]
Hypertension	*Derived from prevalence*	*Health Survey for England 2012* [29]	*non terminal*	*non terminal*	*World Obesity Federation (DYNAMO project)* [24]	*Sullivan et al. 2011* [30]	*Netherlands data as proxy Polder et al, 2002* [31]
Diabetes	*Personal communication Dr. Craig Currie at Cardiff University*	*International Diabetes Federation, 2014* [32]	*non terminal*	*non terminal*	*Derived from PREVEND data (Jaccard 2015 et al. Manuscript in preparation.*	*Sullivan et al. 2011* [30]	*IDF atlas 2014* [32]
Knee Osteoarthritis	*Derived from prevalence*	*Arthritis UK Musculoskeletal calculator* [33]	*non terminal*	*non terminal*	*Zheng et al (2015)* [34]	*Conner-Spady et al. 2015* [35]	*Oxford Economics Report 2010* [36]
Breast cancer	*CRUK, 2013 Statistics by cancer type* [37]	*NA*	*CRUK Mortality by cancer type*[38]	*ONS Cancer Survival in England: adults diagnosed between 2009 and 2013 and followed up to 2014* [39] *& ONS Cancer Survival in England: 10 year survival rates adults diagnosed between 2010-2011 and followed up to 2012* [40]	*World Obesity Federation (DYNAMO project)* [24]	*Sullivan et al. 2011* [30]	*NHS 2012-13 programme budgeting data* [26]
Colorectal cancer		*NA*			*World Obesity Federation (DYNAMO project)* [24]	*Sullivan et al. 2011* [30]	
Endometrial cancer		*NA*			*World Obesity Federation (DYNAMO project)* [24]	*Sullivan et al. 2011* [30]	
Oesophageal cancer		*NA*			*World Obesity Federation (DYNAMO project)* [24]	*Sullivan et al. 2011* [30]	
Ovarian cancer		*NA*			*Aune et al. 2015* [41]	*Sullivan et al. 2011* [30]	
Pancreatic cancer		*NA*			*World Cancer Research Fund, 2007*[42]	*Romanus et al. 2012* [43]	
Renal cancer		*NA*			*World Obesity Federation (DYNAMO project)* [24]	*Sullivan et al. 2011* [30]	

4.2 Results

Mean (SD) BMI change between baseline and 1 year was -1·20 kg/m^2 (2·3), -1·70 kg/m^2 (2·3) and -2·44 kg/m^2 (2·7) for the brief intervention, 12-week programme and 52-week programme respectively; change in BMI between 1 and 2 years was 0·36 kg/m^2 (1·4), 0·62 kg/m^2 (1·3) and 0·89 kg/m^2 (1·6) respectively. Long-term microsimulation modelling estimated that over 25 years after WRAP baseline (by 2039) the 52-week programme would result in a lower incidence of disease, fewer direct healthcare costs of diseases, and more QALYs than the brief intervention or 12-week programme.

Compared to the 12-week programme, the 52-week programme resulted in 1786 fewer incident cases of the diseases of interest per 100,000 individuals, including 642 fewer cases of hypertension, 373 fewer cases of diabetes and 104 fewer cases of CHD for every 100,000 individuals (Figure A4). This resulted in £8·61 million in direct healthcare costs avoided per 100,000 individuals (Figure A5). Taking into account intervention costs, the 52-week programme was cost-effective, compared to the 12-week intervention, resulting in 1282 additional QALYs per 100,000 individuals (Figure A6) at a cost of £4·9 million per 100,000 individuals. The ICER (£3804/QALY) indicated that the 52-week programme was cost-effective compared to the 12-week programme for the 2015 to 2039 period.

Compared to the brief intervention, the 52-week programme resulted in an estimated 2409 fewer cases of incident disease per 100,000 individuals in the population, £13·0million avoided in direct healthcare costs per 100,000 individuals. Taking into account intervention costs, the 52-week programme resulted in 1925 additional QALYs gained per 100,000 individuals at a cost of £4·6million per 100,000 individuals. The ICER (£2394/QALY) indicated that the 52-week programme was cost-effective compared to the brief intervention for the 2015 to 2039 period.

Compared to the brief intervention, the 12-week programme avoided 623 incident cases of all diseases modelled and £4·41 million in direct healthcare costs. Taking intervention costs into account, the ICER for the 12-week programme was dominant in comparison to the brief intervention for the period 2015-2039, resulting in 643 additional QALYs per 100,000 individuals, at a cost-saving of £268,000 per 100,000 individuals.

Figure A4 - Total cumulative incidence (+95%CL) avoided per 100,000 by year (2014 to 2039). Outputs for each comparison pair.

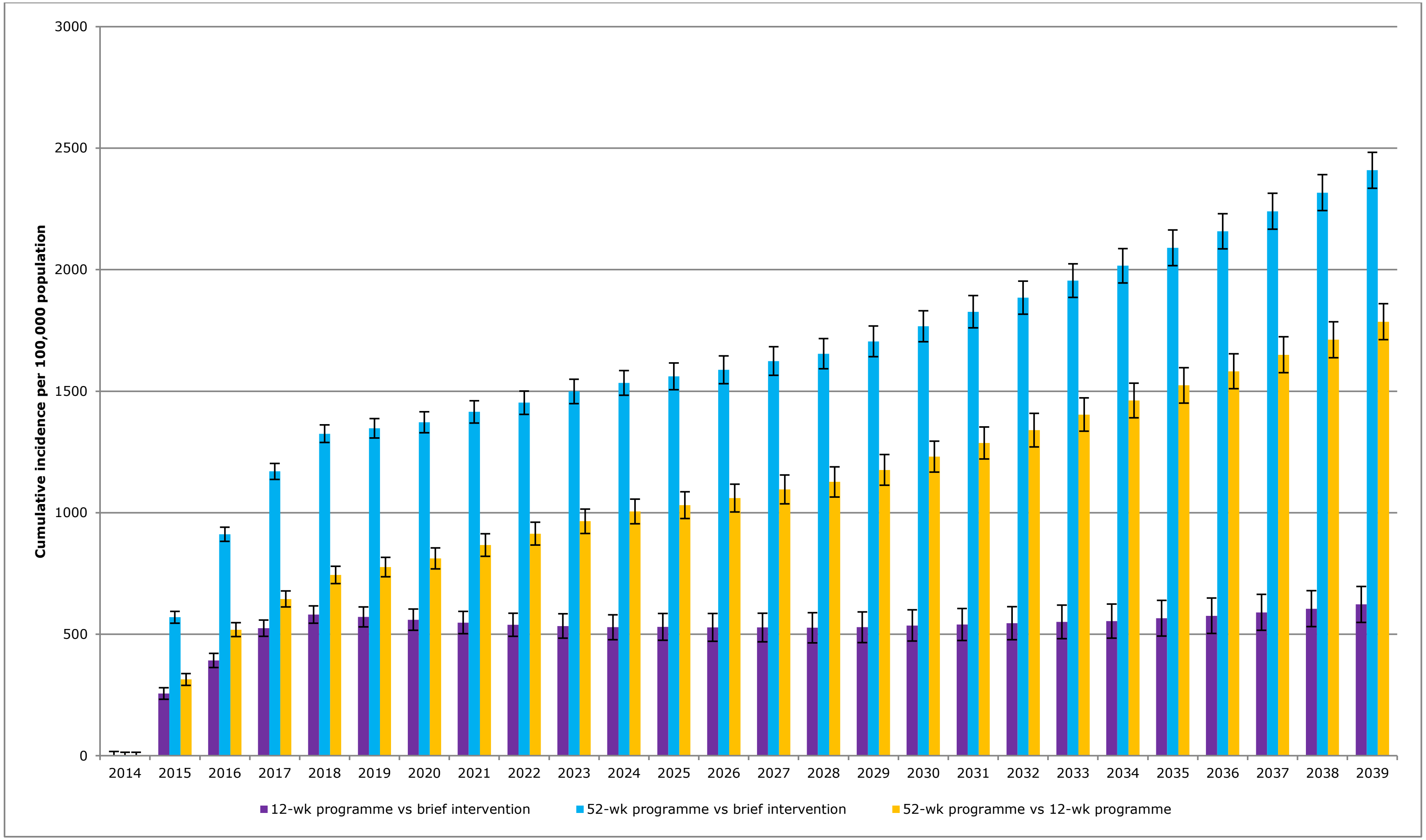

Figure A5 - Cumulative total direct healthcare costs in £Millions (+95%CL) avoided per 100,000 by year (2014 to 2039).
Outputs for each comparison pair.

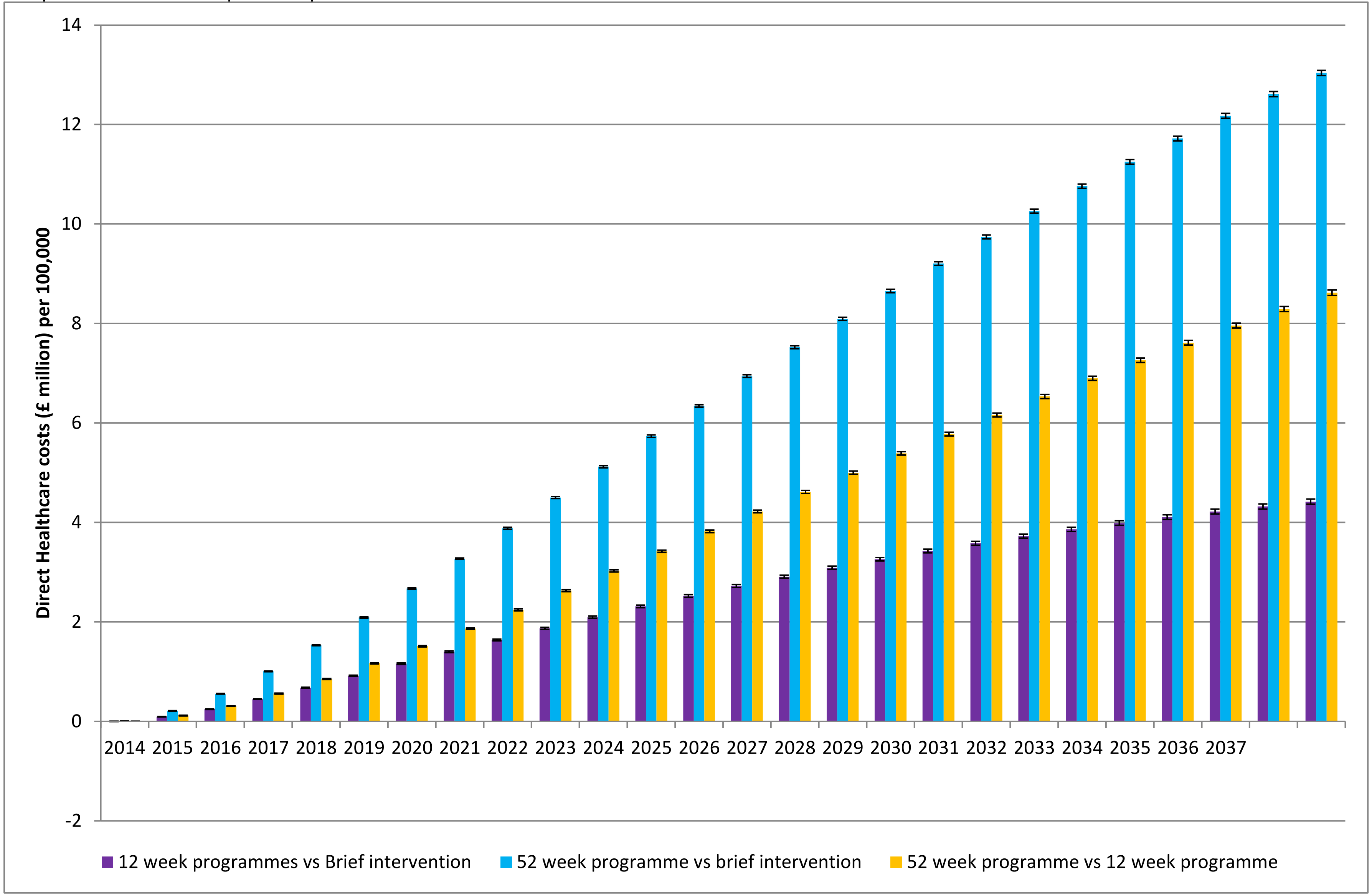

Figure A6 – Cumulative Quality Adjusted Life Years generated annually (+95%CL) per 100,000 by year (2014 to 2039).
Outputs for each comparison pair.

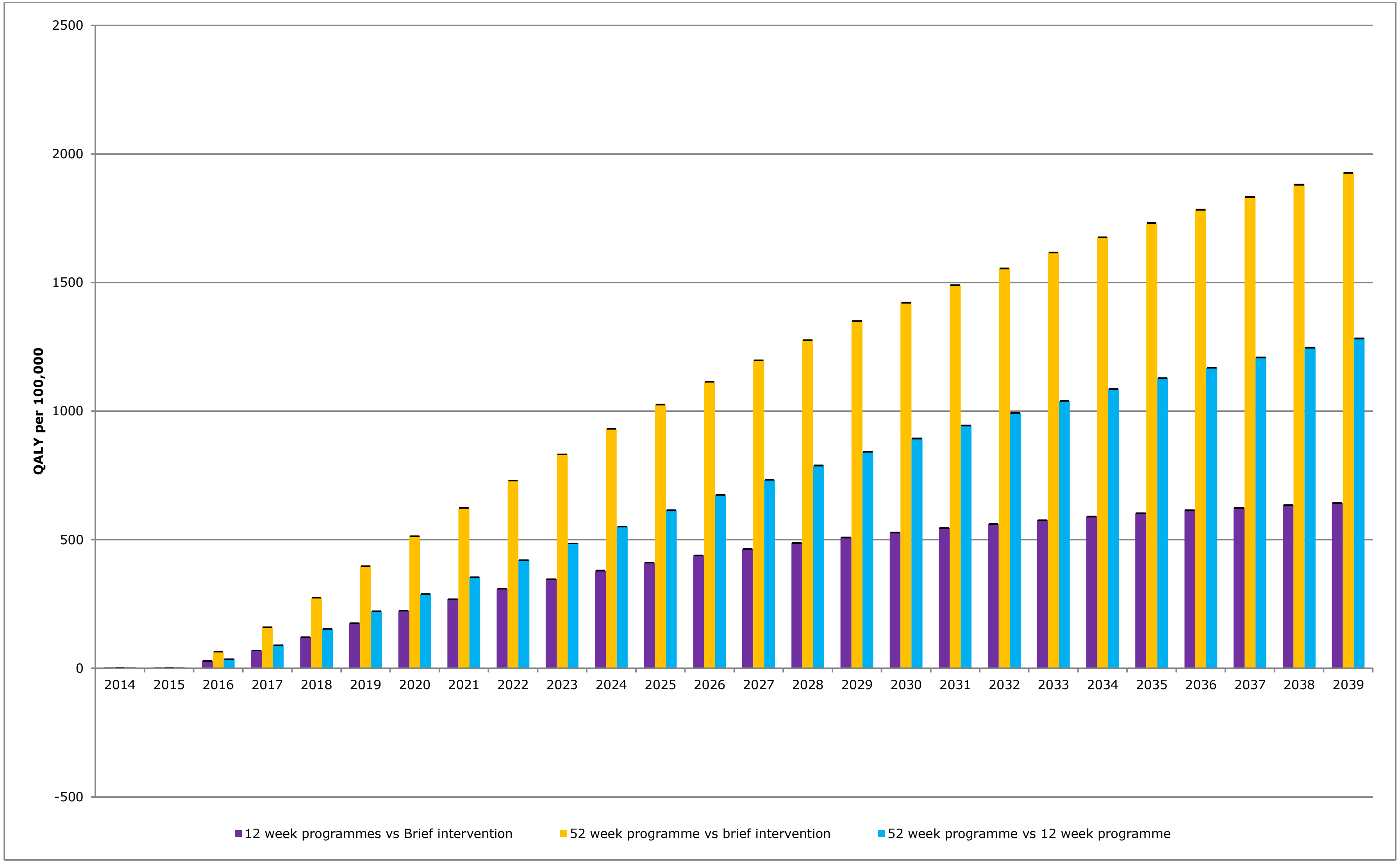

5. References

1. National Institute for Health and Care Excellence (NICE). Guide to the methods of technology appraisal. London; 2013.
2. Hunt K, Wyke S, Gray CM, et al. A gender-sensitised weight loss and healthy living programme for overweight and obese men delivered by Scottish Premier League football clubs (FFIT): a pragmatic randomised controlled trial. *The Lancet* 2014; **383**(9924): 1211-21.
3. Curtis L. Unit costs of health and social care. The University of Kent: Personal Social Services Research Unit; 2014.
4. Department of Health. NHS Schedule of Reference Costs 2013-2014. 2014.
5. Health and Social Care Information Service. Prescription Cost Analysis, England - 2014. 2014.
6. Drummond Michael F. et al. Methods for the economic evaluation of health care programmes. . Oxford university press; 2015.
7. White IR, Royston P, Wood AM. Multiple imputation using chained equations: issues and guidance for practice. *Statistics in medicine* 2011; **30**(4): 377-99.
8. Löthgren M, Zethraeus N. Definition, interpretation and calculation of cost-effectiveness acceptability curves. *Health economics* 2000; **9**(7): 623-30.
9. Jebb SA, Ahern AL, Olson AD, et al. Primary care referral to a commercial provider for weight loss treatment versus standard care: a randomised controlled trial. *The Lancet* 2011; **378**(9801): 1485-92.
10. Fuller N, Colagiuri S, Schofield D, et al. A within-trial cost-effectiveness analysis of primary care referral to a commercial provider for weight loss treatment, relative to standard care—an international randomised controlled trial. *International Journal of Obesity* 2013; **37**(6): 828-34.
11. Tsai AG, Wadden TA, Volger S, et al. Cost-effectiveness of a primary care intervention to treat obesity. *International Journal of Obesity* 2013; **37**: S31-S7.
12. Diabetes Prevention Program Research Group. The Diabetes Prevention Program (DPP) description of lifestyle intervention. *Diabetes care* 2002; **25**(12): 2165-71.
13. Gustafson A, Khavjou O, Stearns SC, et al. Cost-effectiveness of a behavioral weight loss intervention for low-income women: the Weight-Wise Program. *Preventive medicine* 2009; **49**(5): 390-5.
14. Jakicic JM, Tate DF, Lang W, et al. Effect of a stepped-care intervention approach on weight loss in adults: a randomized clinical trial. *JAMA* 2012; **307**(24): 2617-26.
15. Krukowski RA, Tilford JM, Harvey-Berino J, West DS. Comparing Behavioral Weight Loss Modalities: Incremental Cost-Effectiveness of an Internet-Based Versus an In-Person Condition. *Obesity* 2011; **19**(8): 1629-35.
16. Hersey JC, Khavjou O, Strange LB, et al. The efficacy and cost-effectiveness of a community weight management intervention: a randomized controlled trial of the health weight management demonstration. *Preventive medicine* 2012; **54**(1): 42-9.
17. Little P, Stuart B, Hobbs FR, et al. An internet-based intervention with brief nurse support to manage obesity in primary care (POWeR+): a pragmatic, parallel-group, randomised controlled trial. *The Lancet Diabetes & Endocrinology* 2016; **4**(10): 821-8.
18. Hollingworth W, Hawkins J, Lawlor D, Brown M, Marsh T, Kipping R. Economic evaluation of lifestyle interventions to treat overweight or obesity in children. *International Journal of Obesity* 2012; **36**(4): 559-66.
19. McPherson K, Marsh T, Brown M. Tackling obesities: future choices: Modelling future trends in obesity and the impact on health: Citeseer; 2007.
20. NatCen Social Research. Health Survey for England 2003-2016. UK Data Service. https://discover.ukdataservice.ac.uk/series/?sn=2000021.
21. Smolina K, Wright FL, Rayner M, Goldacre MJ. Determinants of the decline in mortality from acute myocardial infarction in England between 2002 and 2010: linked national database study. Corrected data on incidence and mortality in 2013 at http://www.bmj.com/content/347/bmj.f7379.abstract. *BMJ* 2012; **344**: d8059.
22. British Heart Foundation. Cardiovascular Disease Statistics 2014, 2015.
23. Office for National Statistics. Deaths Registrations Summary Statistics, England and Wales, 2014.

24. World Obesity Federation. Relative risk Assessments IASO; Prepared for DYNAMO-HIA project. http://www.worldobesity.org/site_media/uploads/Appendix_Relative_Risk_Assessments_IASO.pdf; http://www.worldobesity.org/what-we-do/policy-prevention/projects/eu-projects/dynamohiaproject/estimatesrrperunitbmi/.
25. Laires PA, Ejzykowicz F, Hsu TY, Ambegaonkar B, Davies G. Cost-effectiveness of adding ezetimibe to atorvastatin vs switching to rosuvastatin therapy in Portugal. *J Med Econ* 2015; **18**(8): 565-72.
26. National Health Service. 2012-13 programme budgeting PCT benchmarking tool, 2013.
27. British Heart Foundation. Stroke Statistics 2009, 2009.
28. Rivero-Arias O, Ouellet M, Gray A, Wolstenholme J, Rothwell PM, Luengo-Fernandez R. Mapping the modified Rankin scale (mRS) measurement into the generic EuroQol (EQ-5D) health outcome. *Med Decis Making* 2010; **30**(3): 341-54.
29. Health and Social Care Information Centre. Health Survey for England 2012. 2012.
30. Sullivan PW, Slejko JF, Sculpher MJ, Ghushchyan V. Catalogue of EQ-5D scores for the United Kingdom. *Med Decis Making* 2011; **31**(6): 800-4.
31. Polder JJ, Bonneux L, Meerding WJ, van der Maas PJ. Age-specific increases in health care costs. *European journal of public health* 2002; **12**(1): 57-62.
32. International Diabetes Federation. Diabetes Atlas, 2014.
33. Arthritis Research UK. Musculoskeletal Calculator. 2016. http://www.arthritisresearchuk.org/arthritis-information/data-and-statistics/musculoskeletal-calculator.aspx.
34. Zheng H, Chen C. Body mass index and risk of knee osteoarthritis: systematic review and meta-analysis of prospective studies. *BMJ open* 2015; **5**(12): e007568.
35. Conner-Spady BL, Marshall DA, Bohm E, et al. Reliability and validity of the EQ-5D-5L compared to the EQ-5D-3L in patients with osteoarthritis referred for hip and knee replacement. *Qual Life Res* 2015; **24**(7): 1775-84.
36. Oxford Economics. The economic cost of arthritis for the UK economy - Final report, 2010.
37. Cancer Research UK. Statistics by cancer type - Average Number of New Cases Per Year and Age-Specific Incidence Rates per 100,000 Population, UK 2011-2013 2016. http://www.cancerresearchuk.org/health-professional/cancer-statistics/statistics-by-cancer-type.
38. Cancer Research UK. Statistics by cancer type - Average Number of Deaths per Year and Age-Specific Mortality Rates, UK, 2010-2012. 2016. http://www.cancerresearchuk.org/health-professional/cancer-statistics/statistics-by-cancer-type.
39. Office for National Statistics. Cancer Survival in England- Adults Diagnosed: 2009 to 2013, followed up to 2014, 2015.
40. Office for National Statistics. Cancer Survival in England: 10 year survival rates adults diagnosed between 2010-2011 and followed up to 2012, 2013.
41. Aune D, Navarro Rosenblatt DA, Chan DSM, et al. Anthropometric factors and ovarian cancer risk: A systematic review and nonlinear dose-response meta-analysis of prospective studies. *International Journal of Cancer* 2015; **136**(8): 1888-98.
42. World Cancer Research Fund. Food, Nutrition, Physical Activity, and the Prevention of Cancer: A Global Perspective. , 2007.
43. Romanus D, Kindler HL, Archer L, et al. Does health-related quality of life improve for advanced pancreatic cancer patients who respond to gemcitabine? Analysis of a randomized phase III trial of the cancer and leukemia group B (CALGB 80303). *J Pain Symptom Manage* 2012; **43**(2): 205-17.
